# *Moraxella salivae* sp. nov., isolated from the saliva of a healthy human volunteer

**DOI:** 10.1080/20002297.2026.2737704

**Published:** 2026-09-23

**Authors:** Qianli Wu, Yuhan Meng, Haoyang Liu, Yali Kong, Yi Chen, Mingyang Bu, Zhe Ma

**Affiliations:** a Department of Preventive Dentistry, School and Hospital of Stomatology, Hebei Medical University, Shijiazhuang, Hebei, China; b Hebei Key Laboratory of Stomatology, Shijiazhuang, Hebei, China; c Hebei Clinical Research Center for Oral Diseases, Shijiazhuang, Hebei, China

**Keywords:** Oral microbiome, novel species, oral saliva, polyphasic taxonomy, phylogenomy

## Abstract

**Background:**

Strain I5G^T^ was isolated from the saliva of a healthy volunteer and polyphasic taxonomic approaches were applied for its systematic identification to clarify its taxonomic position within the genus *Moraxella*.

**Methods:**

A polyphasic taxonomic study was performed, including morphological observation, physiological and biochemical characterisation, chemotaxonomic analyses (fatty acid profile, respiratory quinone, polar lipids), 16S rRNA gene phylogeny, and whole-genome-based phylogenetic and comparative genomic analyses.

**Results:**

Strain I5G^T^ is a Gram-stain-negative, aerobic, rod-shaped bacterium. Growth occurs at 25–37 °C (optimum 37 °C), pH 7.0–9.0 (optimum pH 8.0), and 0–1.0% (w/v) NaCl (optimum 0.5%). 16S rRNA gene analysis placed it in the genus *Moraxella*, with highest similarity (96.17%) to *Moraxella oblonga* IAM 14971ᵀ. Whole-genome phylogeny supported a distinct novel lineage. The maximum average nucleotide identity (ANI) (85.9%) and the highest digital DNA-DNA hybridisation (dDDH) (24.8%) values were well below species thresholds. DNA G + C content was 37.5 mol%. Major fatty acids were C₁₈:₁ ω9c, C₁₆:₀, C₁₈:₀ and Summed Feature 3 (C₁₆:₁ ω7c and/or C₁₆:₁ ω6c), major quinone was CoQ-8, major polar lipids were phosphatidylglycerol, diphosphatidylglycerol and phosphatidylethanolamine.

**Conclusion:**

Strain I5G^T^ represents a novel species within the genus *Moraxella*, for which the name *Moraxella salivae* sp. nov. is proposed. The type strain is I5G^T^ (= KCTC 18404ᵀ = GDMCC 1.5953ᵀ).

## Introduction

*Moraxella* is a genus of Gram-negative, aerobic bacteria belonging to the family Moraxellaceae [1]. As of the present study, the genus *Moraxella* currently comprises 28 species with valid taxonomic names [2]. Cells of this genus are spherical or rod‑shaped. The major fatty acid is C₁₈:₁ ω9c, the predominant respiratory quinone is CoQ-8, and the main polar lipids comprise phosphatidylglycerol, phosphatidylethanolamine, cardiolipin (diphosphatidylglycerol) and monophosphatidylglycerol [3–9].

Several members of the genus *Moraxella* act as significant opportunistic pathogens in humans. For example, *Moraxella catarrhalis*, *Moraxella nonliquefaciens* and *Moraxella osloensis* are closely linked to diverse human infections such as pneumonia, otitis media, meningitis, bacteremia, endophthalmitis and endocarditis [10–17]. The genus also contains several animal pathogens, including *Moraxella bovis*, *Moraxella ovis* and *Moraxella lacunata*, which are responsible for keratoconjunctivitis, corneal ulcers and lung or corneal abscesses [18–22].

Beyond the clearly defined infectious diseases mentioned above, the oral cavity serves as the primary colonisation niche for multiple *Moraxella* species, which are common constituents of the oral microbiota in healthy humans [23]. Published studies have documented cases of opportunistic infections triggered by oral-colonising *Moraxella* species under conditions of compromised host immunity and disrupted oral mucosal barriers. For instance, *Moraxella catarrhalis* participates in mixed infections associated with dental caries and tonsillitis in children [24]; *Moraxella osloensis* has been isolated from blood samples of patients with bacteraemia following tooth extraction, indicating that invasive dental procedures may act as one of the predisposing factors for bloodstream invasion by this species [25].

A Gram‑negative, rod‑shaped bacterium was recovered from the saliva of a healthy human volunteer in January 2025 at the Stomatological Hospital of Hebei Medical University, Shijiazhuang, Hebei Province, China (114°28′42.996″E, 38°2′29.004″N). To preliminarily clarify the taxonomic background of this strain, its full‑length 16S rRNA gene sequence was aligned against the expanded Human Oral Microbiome Database (eHOMD). The sequence similarity between this strain and all cultured closely related oral *Moraxella* species in the database was lower than 95.2%, which failed to meet the conventional species delineation threshold. Nevertheless, a total of 74 uncultured clone sequences sharing greater than 99% sequence identity with this strain were retrieved from GenBank, and these clones were derived from oral tissues including periodontal abscesses and subgingival plaques as well as human skin samples (detailed in Table S1). The sequence with the highest identity of 99.71% has accession number MF578820.1 and was isolated from a human periodontal abscess specimen. Notably, these short partial clone sequences can only confirm that phylogenetically related lineages of this taxon are detectable in human oral samples and cannot independently verify stable long‑term oral colonisation of such lineages. No cultivable strains or validly published species matching these clones are currently available and systematic taxonomic evidence remains lacking.

Therefore, to elucidate the taxonomic position of strain I5G^T^, we performed an in-depth analysis of its phenotypic and genotypic characteristics. Since only a single isolate was obtained in the present study, we cannot evaluate its overall colonisation rate in human populations or its clinical relevance. These questions remain to be addressed in dedicated follow-up investigations.

## Methods

### Isolation and ecology

Saliva sample in this study was collected from a healthy male volunteer aged 20‑50 years. The subject had no systemic diseases and no smoking history. No antibiotics or microecology‑modulating drugs were used within three months before sampling. The subject had no acute infection or trauma and had not received recent dental treatment. The subject exhibited good oral hygiene with healthy periodontal and dental tissues and was free of dental caries, periodontal lesions and oral mucosal diseases. The volunteer fasted for 2 h before sampling and avoided alcohol consumption tooth‑brushing and mouth‑rinsing. A total of 3.0 mL of un‑stimulated saliva was collected using a disposable saliva collector. Salivary microorganisms were isolated using a self-designed in-situ oral microbial cultivation device. Briefly, saliva samples were inoculated into the device and incubated intraorally in situ for 5 days. After incubation, the bacterial suspension was collected via elution. The suspensions were inoculated onto blood agar plates (HKM) by quadrant streaking and incubated aerobically at 37 °C. Colony development was inspected every 24 h. Single colonies were purified through three consecutive passages and subjected to preliminary identification based on 16S rRNA gene sequencing. Strain I5Gᵀ was isolated using this procedure. Full engineering information covering device structure and assembly specifications will be described in detail in an independent methodological article. For long-term preservation, colonies were suspended in nutrient broth (Hopebio) supplemented with 5% (v/v) glycerol and preserved at −80 °C. Strain I5G^T^ has been deposited in the Korean Collection for Type Cultures (KCTC) and Guangdong Microbial Culture Collection Centre (GDMCC) under the accession numbers KCTC 18404ᵀ and GDMCC 1.5953ᵀ, respectively.

### Phylogenetic analysis

To determine levels of 16S rRNA gene sequence identity and phylogenetic relationship of strain I5G^T^, genomic DNA was isolated with the TIANamp Bacterial DNA Kit (Tiangen-Biotech) according to the manufacturer’s protocol. The almost complete 16S rRNA gene was amplified using the universal primers 27F and 1492R [26]. The amplified fragment was purified via the AxyPrep DNA Gel Extraction Kit and analysed using an ABI 3730-XL DNA sequencer. Sequence similarities between strain I5G^T^ and closely related taxa were calculated using the EzTaxon-e database [27]. Multiple sequence alignments were carried out with Clustal W. The final alignment comprised 1,265 positions, of which 102 were parsimony-informative sites. Phylogenetic trees were constructed in MEGA 11.0 using the Neighbour-Joining (NJ) [28], Minimum Evolution (ME) [29] and Maximum Likelihood (ML) [30] algorithms. Bootstrap support values were calculated from 1000 replicates and values ≥ 50% are shown at branch nodes. For NJ and ME analyses, maximum composite likelihood model was used with complete deletion of gaps. For ML phylogenetic reconstruction, the Tamura-Nei (TN93) model was determined as the optimal nucleotide substitution model via model testing and was used for tree building.

### Morphological, physiological and biochemical analyses

Gram staining was conducted using a commercial kit (Solarbio), and cellular morphology was observed using an Olympus CX31 light microscope. For scanning electron microscopy, cells grown on Columbia agar (HKM) at 37 °C for 3 days were examined. Anaerobic growth was assessed daily on Columbia agar at 28 °C in an anaerobic chamber with an anaerobic detection kit (Oxoid). Motility was determined using the semi-solid agar stab method with Columbia agar [31]. Growth temperature range and optimum were assessed on Columbia agar at 0, 4, 10, 15, 20, 25, 30, 35, 37, 40 and 45 °C for 72 h. The pH range and optimal pH for growth were determined in nutrient broth (Hopebio) adjusted to pH 4.0–10.0 at 1.0 unit intervals at 37 °C for 72 h, using the buffer system reported by Lee et al. [32]. NaCl tolerance and optimal concentration were assessed in nutrient broth containing 0–5.0% (w/v) NaCl at 0.5% steps, based on optical density readings at 540 nm. Growth on MacConkey agar (Hopebio) was evaluated after 72 h incubation at 37 °C, and haemolytic activity was determined under the same incubation conditions on 5% (v/v) sheep blood agar (HKM). Catalase activity was assessed using 3% (v/v) hydrogen peroxide solution, and oxidase activity was tested with an oxidase reagent (BioMérieux) following the manufacturer’s protocol. Additional biochemical features were characterised using API 20E, API 20NE (assimilation tests incubated for 72 h) and API ZYM strips (BioMérieux). All tests were carried out in three biological replicates under identical conditions.

Antimicrobial susceptibility testing of strain I5Gᵀ was performed via the Kirby-Bauer disk diffusion method on Mueller-Hinton agar plates (BECKMAN) following the guidelines issued by the Clinical and Laboratory Standards Institute (CLSI). The bacterial suspension was adjusted to 0.5 McFarland for inoculum standardisation. Inoculation was performed on MH agar plates using the spread‑plate method. Plates were incubated at 35 °C with 5% CO₂ for 20–24 h and *Staphylococcus aureus* ATCC 25923 was used as the quality‑control strain. The tested antimicrobial discs (BECKMAN) included doxycycline (DO, 30 μg), gentamicin (GEN, 10 μg), penicillin G (PEN, 10 μg), amoxicillin-clavulanate (AMC, 20/10 μg), ciprofloxacin (CIP, 5 μg), ceftriaxone (CTR, 30 μg), levofloxacin (LEV, 5 μg), ampicillin (AMP, 10 μg), cefuroxime (CXM, 30 μg), amoxicillin (AMX, 25 μg), azithromycin (AZI, 15 μg) and metronidazole (MTZ, 5 μg). Since breakpoints for antimicrobial susceptibility have not been established for any *Moraxella* species other than *Moraxella catarrhalis*, strain susceptibility results were interpreted in accordance with the CLSI standards specified for *M. catarrhalis* [33].

### Genomic analysis

Genomic DNA was isolated from exponentially growing cells using a modified SDS lysis method combined with purification columns [34]. The NanoDrop One spectrophotometer (NanoDrop Technologies) and the Qubit 3.0 fluorometer (Life Technologies) were used to measure DNA concentration, and DNA integrity and purity were assessed by agarose gel electrophoresis [35]. A sequencing library was prepared using the VAHTS® Universal Plus DNA Library Prep Kit for Illumina V2 (Vazyme) [36]. In brief, 100 pg to 1 µg of genomic DNA was fragmented, followed by end repair, 5′-phosphorylation and 3′-dA-tailing. Adaptors were then ligated to both ends of the DNA fragments. Two rounds of magnetic bead-based size selection (0.56 × and 0.2 × ratios) were carried out using VAHTS DNA Clean Beads to enrich the target fragment size. The library was amplified using a high-fidelity DNA polymerase. The constructed library was first quantified with a Qubit fluorometer (Thermo Fisher Scientific), and the insert size was verified using the NGS3K/Caliper system (PerkinElmer). For libraries with the expected insert size, the effective concentration (3 nM) was accurately determined by qPCR. Paired-end 150 bp sequencing was subsequently performed on the Illumina NovaSeq 6000 platform [37]. The genome was assembled into a whole-genome shotgun (WGS) draft using Unicycler [38]. The average sequencing depth was 612.15×, and the final assembly yielded 57 contigs. Assembly quality was assessed based on contig statistics and N50 value. Genome completeness and contamination were assessed using CheckM2 (v. 1.1.0). Protein-coding genes were predicted from the assembled genome using Prokka (v. 1.14.6) [39]. Pseudogene candidates were screened using Pseudofinder (v. 1.1.0) based on GenBank files after genome annotation [40]. Functional annotation was achieved by aligning predicted genes against the COG, KEGG, Uniprot and RefSeq databases using BLAST+ (v. 2.11.0+) [41]. Further functional classification was performed using hmmscan (v. 3.3.2) [42] against the Pfam and TIGERFAM databases. Phylogenetic relationships based on whole-genome sequences were inferred via the Type (Strain) Genome Server (TYGS). Genomic data from strain I5G^T^ and its phylogenetically related strains were submitted to the TYGS web server [43]. Phylogenetic trees were generated from distance matrices using the Genome BLAST Distance Phylogeny (GBDP) algorithm together with FastME 2.1.6.1 [44]. Branch confidence was assessed with 100 bootstrap replicates, and phylogenetic trees were displayed and annotated using iTOL version 6. The phylogenetic tree was reconstructed using UBCG software (v. 3.0.0) based on 92 bacterial core genes. The UBCG pipeline employed Prodigal (v. 2.6.3) for gene prediction and Hmmsearch (v. 3.3.2) for core gene identification and extraction. The obtained core gene sequences were subjected to multiple sequence alignment using MAFFT (v. 7.505). The aligned sequences were then imported into RAxML (v. 8.2.12) to construct a maximum-likelihood (ML) phylogenetic tree under the GTRCAT nucleotide substitution model, with 1000 bootstrap replicates to assess the support values of each branch node. Average Nucleotide Identity (ANI) was computed with the OrthoANI (OAT) tool provided by ChunLab (https://www.ezbiocloud.net/tools/orthoani). Digital DNA-DNA hybridisation (dDDH) values were estimated using the Genome-to-Genome Distance Calculator (GGDC) service at DSMZ (https://ggdc.dsmz.de/), with GGDC Formula 2 selected for calculation. All pairwise OrthoANI and digital DNA-DNA hybridisation (dDDH) comparisons were performed on genomes screened based on 16S rRNA gene BLAST alignment results from the EzTaxon‑e database and included all validly‑published type strains of the genus *Moraxella* retrieved from BLAST outputs. Detailed genome information is shown in Supplementary Table S2. The virulence genes were annotated by using the Virulence Factors Database (VFDB) with the parameters described by Li et al. [9]. Potential virulence‑associated genes were screened against the VFDB database (release 2022‑03‑11). BLASTp searches were performed with the following cut‑offs: E‑value ≤ 1e‑5, amino‑acid identity ≥ 60%, query coverage ≥ 70%. All significant blast hits satisfying these criteria were recorded and summarised in Supplementary Table S3. Antimicrobial resistance genes were identified using the Comprehensive Antibiotic Resistance Database (CARD) with the Resistance Gene Identifier (RGI) tool under the strict cutoff setting. Functional annotation of the predicted protein-coding genes was performed using the Kyoto Encyclopaedia of Genes and Genomes (KEGG) database. KEGG pathway classification was used to categorise genes into five major functional classes: Metabolism, Genetic Information Processing, Environmental Information Processing, Cellular Processes and Organismal Systems.

### Chemotaxonomic analysis

Analysis of fatty acid profiles was performed for strain I5Gᵀ and three reference type strains, namely *M. oblonga* CCUG 54938ᵀ, *M. nonliquefaciens* CCUG 348ᵀ and *M. nasovis* CCUG 75922ᵀ, at the Guangdong Microbial Culture Collection Centre (GDMCC, Guangzhou, China). All four strains were cultivated and analysed under identical conditions. Fatty acids were extracted, saponified, methylated and analysed using the Sherlock Microbial Identification System version 6.3 (MIDI), and identified using the TSBA 6.0 database [45]. Polar lipids of strain I5G^T^ and the reference strains were determined by thin-layer chromatography (TLC) at GDMCC. Qualitative analysis was performed using a two-dimensional chromatography system with five specific reagents: phosphomolybdic acid, ninhydrin, D reagent, molybdenum blue and 1-naphthol [46]. Respiratory quinones were extracted, purified and determined by high-performance liquid chromatography-mass spectrometry according to a previously described method [47]. Fatty acid, polar lipid and respiratory quinone data for the other four reference type strains, including *M. bovis* CCUG 2133ᵀ, *M. nasicaprae* ZY201224ᵀ, *M. cuniculi* CCUG 2154ᵀ and *M. caviae* CCUG 355ᵀ, were retrieved from previously published literature.

## Results and discussion

### Phylogenetic analysis

Examination of the 16S rRNA gene sequence using EzTaxon and BLAST showed that strain I5G^T^ was most closely related to *M. oblonga* IAM 14971ᵀ (96.17%) and *M. equi* CCUG 4950ᵀ (96.09%). The sequence similarity between the I5G^T^ strain and all other published *Moraxella* species was less than 96.0% (Table S4), which is notably below the widely recognised species delineation threshold of 98.7%. Consequently, a phylogenetic tree was generated based on 16S rRNA gene sequences from all validly described taxa of the genus *Moraxella*. Topologies obtained with different algorithms were highly consistent, with no obvious variation in the phylogenetic placement of strain I5G^T^. In all three trees (Figures 1, S2 and S3), strain I5Gᵀ fell into a monophyletic clade together with several recognised *Moraxella* species within the genus *Moraxella*. This clade received bootstrap support values of 97% in the NJ tree, 94% in the ME tree and 75% in the ML tree, indicating strong support in the NJ and ME analyses and moderate support in the ML analysis. Strain I5Gᵀ did not cluster tightly with any single recognised species, supporting its phylogenetic separation from currently described taxa. Strain I5G^T^ also showed the closest phylogenetic relationship with *M. oblonga* CCUG 54938ᵀ and *M. nonliquefaciens* CCUG 348ᵀ, supporting its placement in the genus *Moraxella* as a novel phylogenetic lineage.

**Figure 1.**
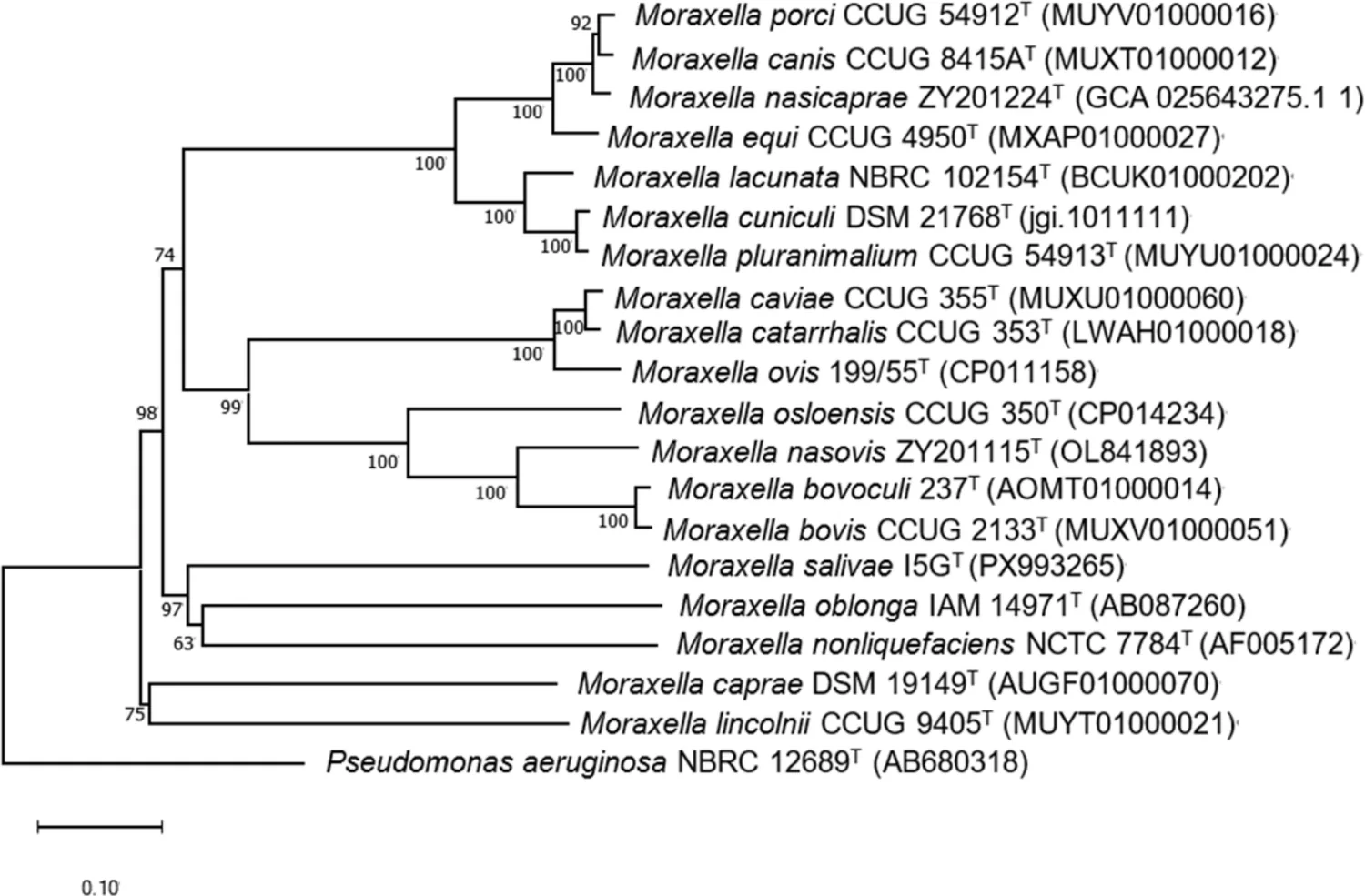
Neighbour-joining phylogenetic tree based on 16S rRNA gene sequences showing the position of strain I5G^T^ among type strains of the genus *Moraxella*. Bootstrap values over 50% are indicated at branch points. *Pseudomonas aeruginosa* NBRC 12689^T^ was used as an outgroup. Bar, 0.10 substitutions per nucleotide position.

### Morphological, physiological and biochemical analyses

Strain I5G^T^ consists of non‑motile, Gram‑negative cells. Scanning electron microscopy shows that cells are rod‑shaped, approximately 1.5–4.0 µm long and 0.4–0.6 µm wide (Figure 2). This morphology is consistent with the genus *Moraxella*, members of which are generally coccoid or rod‑shaped [3]. The strain was able to grow at temperatures between 25 and 37 °C, pH values from 7.0 to 9.0 and NaCl concentrations of 0–1.0% (w/v). The most favourable growth conditions were 37 °C, pH 8.0 and 0.5% (w/v) NaCl. Colonies on sheep blood agar are grayish‑white, opaque, circular and smooth, with a diameter of 1.0–3.0 mm. The main physiological and biochemical characteristics of strain I5Gᵀ are as follows: it produces oxidase, esterase (C4), lipase (C8), leucine arylamidase and naphthol-AS-BI-phosphohydrolase, but lacks *α*-galactosidase, *β*-glucuronidase, N-acetyl-*β*-glucosaminidase, *β*-fucosidase, *β*-galactosidase, arginine dihydrolase, lysine decarboxylase, ornithine decarboxylase, tryptophan deaminase, urease and indole. Oxidative fermentation (OF) tests for D-glucose, D-mannitol, myo-inositol, D-sorbitol, L-rhamnose, sucrose, melibiose, amygdalin and L-arabinose yielded negative results. The strain failed to assimilate D-glucose, L-arabinose, D-mannose, D-mannitol, *N*-acetyl-D-glucosamine, maltose, gluconate, capric acid, adipic acid, malate, citric acid and phenylacetic acid. In addition, the catalase-negative reaction distinguishes this strain from seven reference type strains within the genus *Moraxella* (Table 1). Phenotypic differences distinguish strain I5G^T^ from its closely related reference strains, supporting that strain I5G^T^ represents a novel species within the genus *Moraxella*.

**Figure 2.**
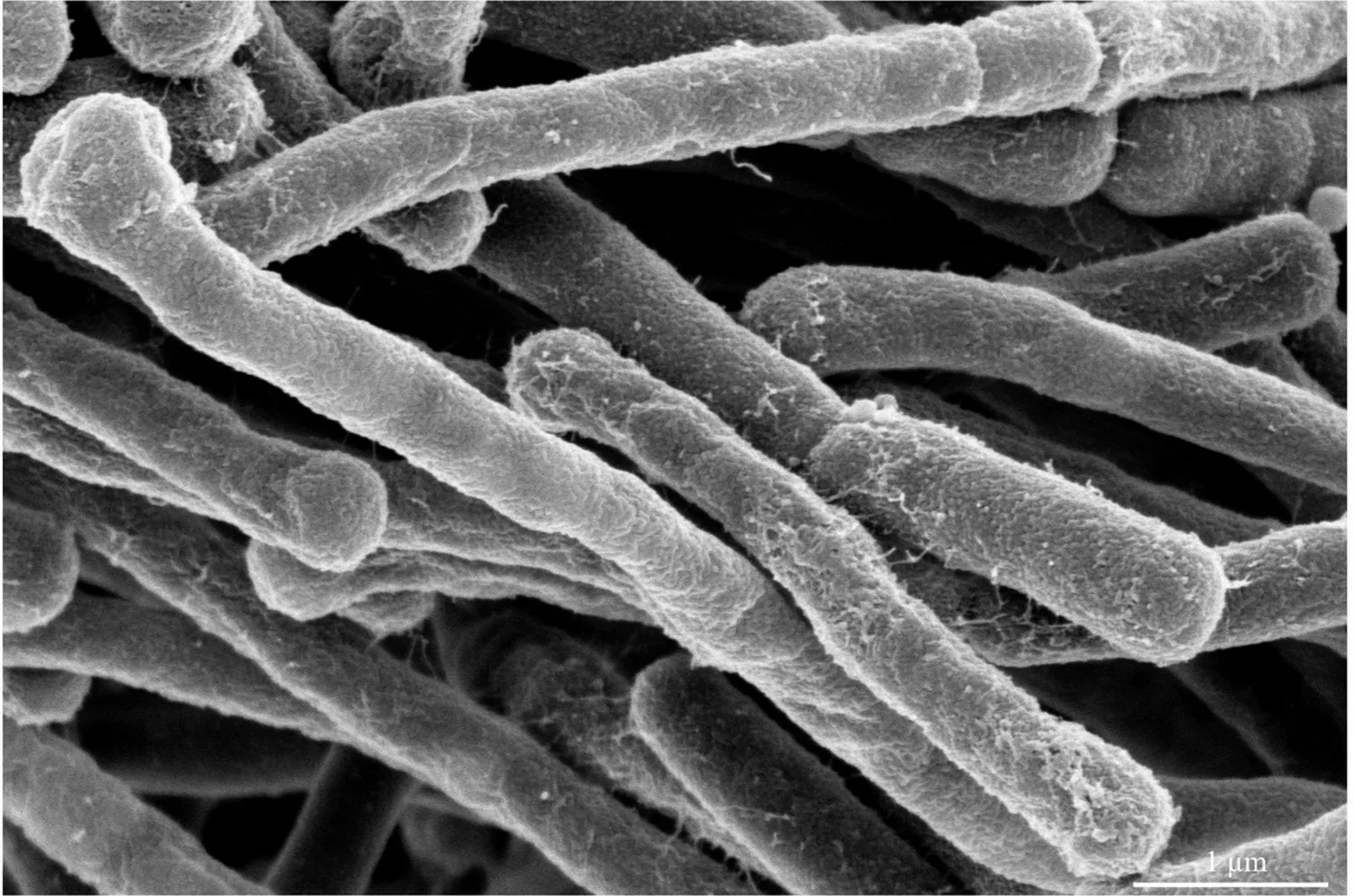
Scanning electron microscope of strain I5G^T^ on Columbia agar at 37 °C for 3 days, showing the rod-shaped cells. The scale bar is 1.0 µm.

**Table 1.** Differential characteristics between strain I5G^T^ and the type strains of its closely related species in the genus *Moraxella.*

Characteristic	1	2	3	4	5*	6*	7*	8*	9*	10*
Activity of										
Esterase (C4)	+	+	+	+	+	+	+	+	+	+
Esterase lipase (C8)	+	+	+	+	+	+	+	+	+	+
Naphthol-AS-BI-phosphohydrolase	+	+	+	+	+	+	−	+	+	+
Leucine arylamidase	+	+	+	+	+	+	+	+	+	+
Valine arylamidase	w	+	w	+	−	ND	−	ND	−	−
Acid phosphatase	−	−	w	w	+	+	−	−	−	−
Oxidase	+	+	+	+	+	+	+	+	+	+
Catalase	−	+	+	+	−	+	+	+	+	+
Assimilation of										
Glucose	−	−	w	−	+	+	−	+	−	+
Arabinose	−	−	−	−	−	+	−	+	−	−
Mannose	−	−	−	−	+	+	−	+	−	−
Mannitol	−	−	−	−	+	+	−	−	−	−
* N*-acetyl-glucosamine	−	−	−	−	−	+	−	+	−	−
Maltose	−	−	−	−	−	+	−	+	−	−
Potassium gluconate	−	−	−	−	−	−	−	+	−	−
Trisodium citrate	−	−	w	−	−	−	−	ND	−	−
Adipic acid	−	−	−	−	−	−	−	+	−	−
Malate	−	−	−	−	−	+	+	+	+	+
Phenylacetic acid	−	−	−	−	−	−	−	ND	−	−
Voges-Proskauer test	−	−	−	−	+	−	−	−	−	−
Nitrate reduction	+	−	−	−	−	+	−	+	+	−
Fermentation of glucose	−	−	−	−	−	−	+	+	−	−
Gelatine hydrolysis	w	w	+	w	+	−	−	−	−	−
*α*-Haemolysis	−	−	−	−	−	+	−	−	−	−
Morphology	rod	rod	coc-cus in chains	coccus	rod	coccus	coccus	coccus	coccus	coccus
DNA G + C content (mol%)	37.5	42.0	41.0	42.0	43.9	43.6	44.3	46.5	46.6	46.4

* Data from Li et al. [9,48], Vela et al. [8,49].

+, Positive; –, negative; w, weakly positive; ND, not determined.

Strains: 1, I5G^T^; 2, *Moraxella nonliquefaciens* CCUG 348^T^; 3, *Moraxella oblonga* CCUG 54938^T^; 4, *Moraxella nasovis* CCUG 75922^T^; 5, *Moraxella bovis* CCUG 2133^T^; 6, *Moraxella nasicaprae* ZY201224^T^; 7, *Moraxella cuniculi* CCUG 2154^T^; 8, *Moraxella caviae* CCUG 355^T^; 9, *Moraxella porci* CCUG 54912^T^; 10, *Moraxella pluranimalium* CCUG 54913^T^.

Per CLSI guidelines, official zone diameter breakpoints for the Kirby-Bauer disk diffusion assay of *Moraxella catarrhalis* are only available for three antimicrobials: amoxicillin-clavulanate (AMC), tetracyclines represented by doxycycline (DO), and azithromycin (AZI). These breakpoints were applied in the present study to classify susceptibility phenotypes as susceptible (S), intermediate (I) or resistant (R). It should be noted that these breakpoints have not been validated for strain I5Gᵀ; accordingly, the resulting susceptibility classifications do not represent definitive clinical susceptibility phenotypes for this novel species. The other nine tested antimicrobials with no official CLSI interpretive criteria are listed as follows: penicillin G (PEN), ampicillin (AMP), amoxicillin (AMX), cefuroxime (CXM), ceftriaxone (CTR), ciprofloxacin (CIP), levofloxacin (LEV), gentamicin (GEN) and metronidazole (MTZ). For antimicrobial agents without CLSI interpretive breakpoints for the genus *Moraxella*, zones of inhibition were observed for all tested discs except metronidazole. No zone of inhibition was observed around metronidazole, which is consistent with the intrinsic inactivity of metronidazole against aerobic Gram‑negative bacteria; this observation should not be interpreted as acquired resistance. Zone‑of‑inhibition diameters for the remaining agents without available breakpoints are reported only as raw measured values in Table 2. No susceptible or resistant phenotype was assigned owing to the lack of valid interpretive criteria.

**Table 2.** Inhibition zone diameters (mm) of 12 commercial antibiotic discs against strain I5Gᵀ.

Antibiotic (disc content)	Abbr.	Mean inhibition zone diameter of strain I5Gᵀ (mm)	Susceptibility category
Amoxicillin-clavulanate (20/10 μg)	AMC*	32	S
Doxycycline (30 μg)	DO*	29	S
Azithromycin (15 μg)	AZI*	27	S
Penicillin G (10 μg)	PEN	30	-
Ampicillin (10 μg)	AMP	30	-
Amoxicillin (25 μg)	AMX	30	-
Cefuroxime (30 μg)	CXM	29	-
Ceftriaxone (30 μg)	CTR	29	-
Ciprofloxacin (5 μg)	CIP	30	-
Levofloxacin (5 μg)	LEV	30	-
Gentamicin (10 μg)	GEN	29	-
Metronidazole (5 μg)	MTZ	ND	-

Drugs marked with an asterisk (*) have disk diffusion interpretive breakpoints for *Moraxella catarrhalis* defined by CLSI, allowing classification as susceptible (S), intermediate (I) or resistant (R). The other antimicrobials have no CLSI disk diffusion criteria for the genus *Moraxella*, only raw zone diameters are shown without susceptibility grading. ND, no visible zone of inhibition.

### Genomic analysis

Strain I5G^T^ has a draft genome assembly of 2,084,350 bp, with an N50 of 124,977 and a DNA G + C content of 37.5 mol% (Table 1). A total of 57 contigs were generated in this assembly, with an average sequencing depth of 612.15 × . CheckM2 assessment revealed a genome completeness of 99.99% and contamination of 0.12%, meeting the quality criteria for taxonomic genomic analysis. Genome annotation showed that the strain possessed one set each of 5S rRNA, 16S rRNA and 23S rRNA genes, one tmRNA, eight miscellaneous non-coding RNAs (misc_RNA) and 42 tRNA genes; a total of 1893 protein‑coding sequences (CDSs) and 124 pseudogene candidates were predicted. It should be noted that contig breaks and assembly artefacts at sequence ends may lead to overestimated pseudogene counts for fragmented short‑read assembled genomes. Therefore this pseudogene number only represents a preliminary annotation output and cannot be directly regarded as experimentally validated biological features. A GBDP phylogenetic tree including all related type strains was built using the TYGS platform (Figure 3), revealing that strain I5G^T^ forms a separate lineage within the genus *Moraxella* and is closely grouped with *M. pluranimalium* CCUG 54913ᵀ and *M. porci* CCUG 54912ᵀ. Phylogenetic analysis based on 92 single-copy core genes indicated that strain I5Gᵀ forms an independent, deeply branched monophyletic lineage within the genus *Moraxella*, with a bootstrap support value of 100% (Figure 4). This high-resolution analysis further clarified the distinct phylogenetic position of this strain within the genus. Whole-genome ANI analysis showed that the highest identity value (85.9%) was observed between strain I5G^T^ and *M. bovis* CCUG 2133ᵀ (Table S5), which is notably below the 95% species demarcation threshold [50,51]. Digital DNA-DNA hybridisation (dDDH) analysis indicated a maximum dDDH value of 24.8% between strain I5G^T^ and *M. nasicaprae* ZY201224ᵀ or *M. cuniculi* NCTC 10297ᵀ (Table S6), far below the 70% cut-off for species definition [50]. Multiple homologous matches to VFDB entries were detected (Table S3). Because several of these proteins have broadly conserved cellular functions and their specific roles have not been experimentally validated in strain I5Gᵀ, these sequence matches were not interpreted as evidence of virulence or pathogenicity. One vanY-like homologous match was detected on ctg_00541. This fragment shared 34.68% amino-acid identity with vanY (an auxiliary gene of the vanB glycopeptide-resistance gene cluster). The local alignment spanned beyond the full-length reference VanY protein. No complete open reading frame could be located at this locus. The complete vanB–vanR–vanS operon was not detected in the present draft assembly. This fragment exists in isolation within the genome, and no additional resistance-associated genes were detected in the immediately flanking regions represented on this assembled contig. Combined with the low sequence similarity, this alignment result most likely represents an endogenous peptidase homologue of this bacterium rather than a functional vanY glycopeptide‑resistance determinant. Genome annotation identified antioxidant‑related genes sodA (COG0605) and ahpC‑ahpF (COG0450, COG3634) in strain I5Gᵀ. These genes are widely distributed among the genomes of diverse bacteria. All predicted coding sequences were assigned to five major KEGG functional branches: Metabolism, Genetic Information Processing, Environmental Information Processing, Cellular Processes and Organismal Systems (Figure 5). Genes under the Metabolism category were the most abundant, dominated by global and overview maps (434 genes), metabolism of cofactors and vitamins (128 genes) and amino acid metabolism (108 genes). Environmental Information Processing consisted of 52 signal transduction genes and 42 membrane transport genes. Within Genetic Information Processing, translation-related genes (79 genes) were the most numerous. In Cellular Processes, genes for cellular community­prokaryotes constituted the predominant subcategory.

**Figure 3.**
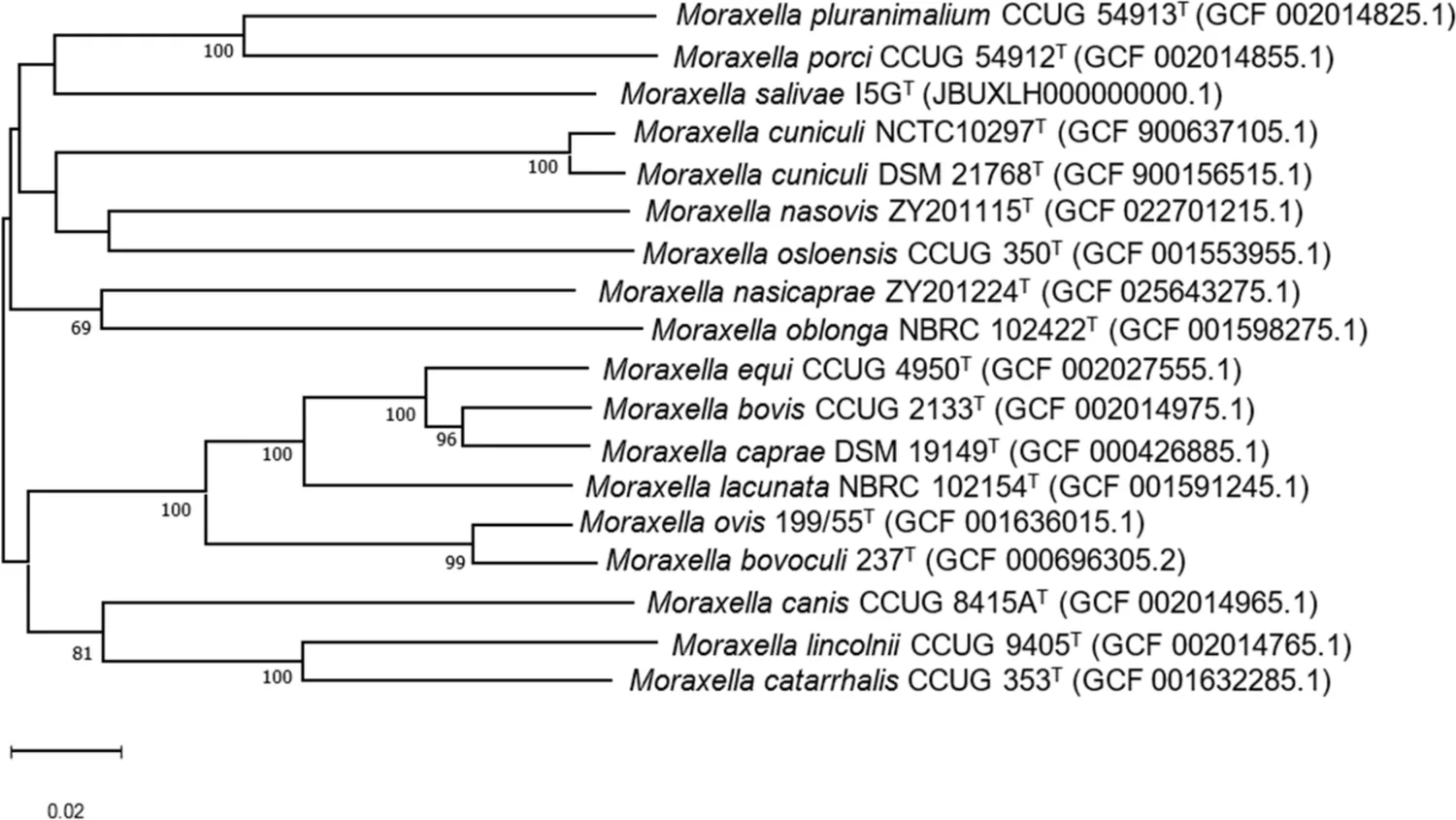
Phylogenetic tree based on whole-genome sequences showing the phylogenetic relationship between strain I5G^T^ and type strains of the genus *Moraxella*. Bootstrap values are indicated at branch points. Bar, 0.02 substitutions per nucleotide position.

**Figure 4.**
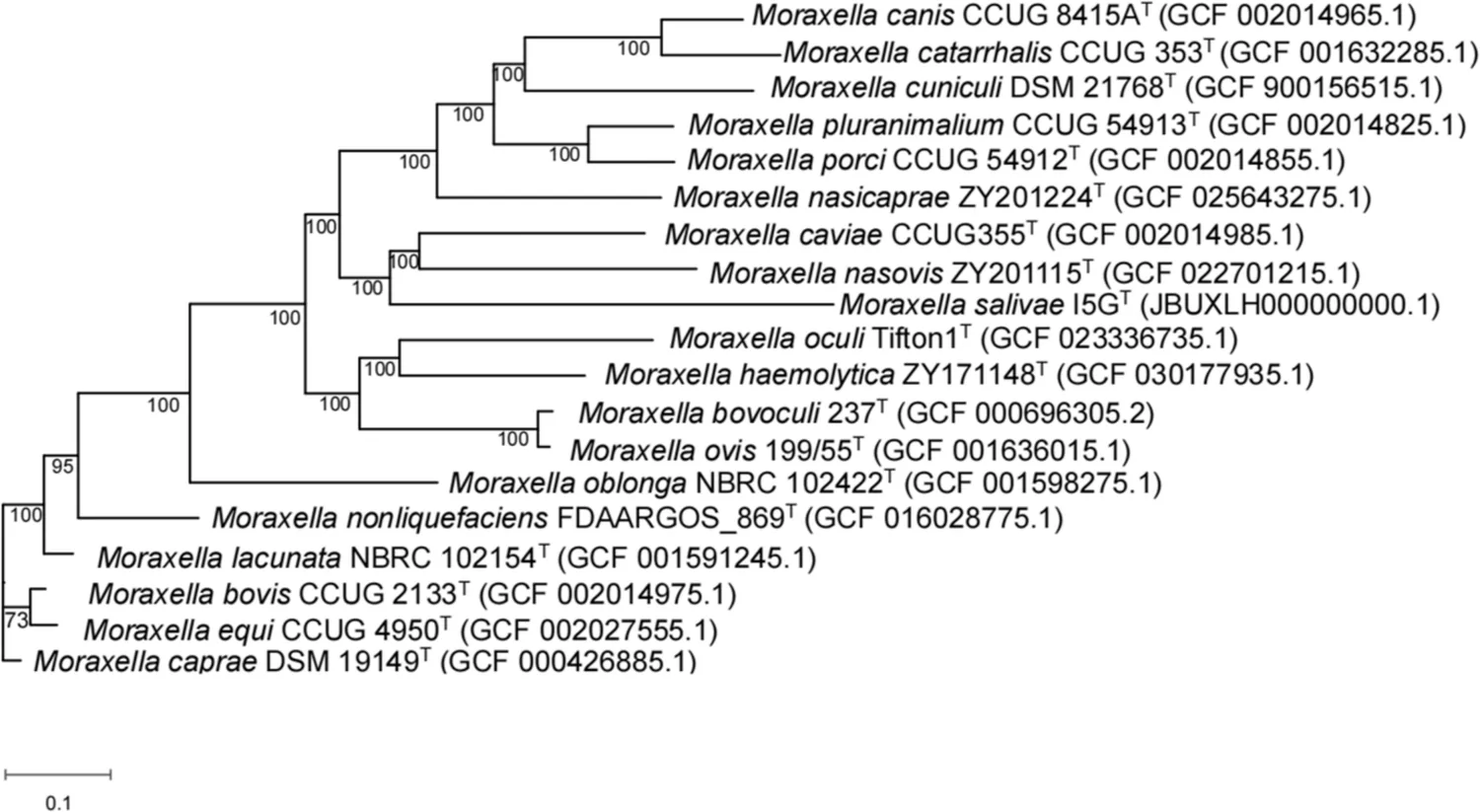
Maximum-likelihood phylogenetic tree of strain I5Gᵀ and related type strains of the genus *Moraxella*, constructed based on the 92 single-copy core-gene set implemented in UBCG. Bootstrap values are indicated at branch points. Bar, 0.1 substitutions per nucleotide position.

**Figure 5.**
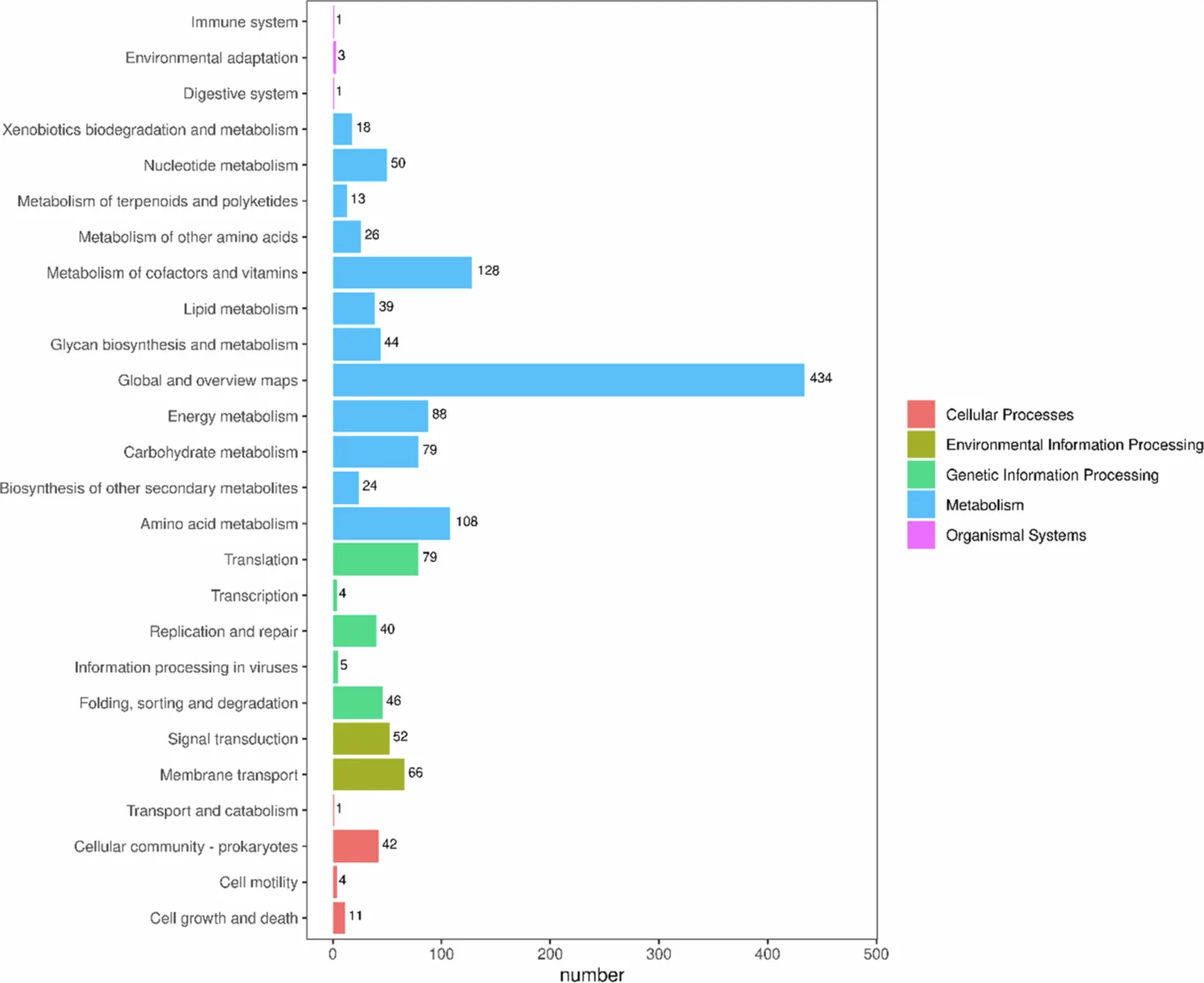
KEGG pathway classification of predicted protein-coding genes of strain I5Gᵀ.

It should be noted that all genomic functional analyses in this study were based on bioinformatic predictions of a single strain. These analyses are not universally applicable to the species, cannot reflect the genetic and phenotypic diversity of this species, and are insufficient to verify the oral colonisation characteristics, ecological functions and clinical relevance of this strain. Since only one isolate obtained from a single individual was included in the present study, it remains unclear whether this taxon represents a persistent oral coloniser, a transient microorganism or an opportunistic isolate. Further studies with expanded sample sizes, cohort investigations, longitudinal monitoring and in‑vitro functional experiments are required to clarify its ecological features and clinical significance.

### Chemotaxonomic analysis

The fatty acid compositions of strain I5Gᵀ and three experimentally tested reference type strains are shown in Table 3, together with comparative data of other reference strains from published literature. The dominant fatty acids of strain I5G^T^ are C₁₈:₁ ω9c, C₁₆:₀, C₁₈:₀ and Summed Feature 3 (C₁₆:₁ ω7c and/or C₁₆:₁ ω6c) (Table 3), which is generally consistent with the chemotaxonomic characteristics reported for the genus *Moraxella* [52,53]. Among strains measured under identical culture conditions, the proportion of C₁₈:₀ in strain I5Gᵀ (12.7%) was higher than those of *M. nonliquefaciens* CCUG 348^T^ (3.0%), *M. oblonga* CCUG 54938^T^ (2.7%) and *M. nasovis* CCUG 75922^T^ (2.2%), and this characteristic can aid phenotypic differentiation of the strain. The major and minor respiratory quinones of strain I5G^T^ are CoQ-8 (Coenzyme Q8, 82.5%) and CoQ-7 (Coenzyme Q7, 12.5%), which are consistent with the five reference strains (Table 3), indicating a close phylogenetic relationship with the genus *Moraxella*. The primary respiratory quinone in the genus *Moraxella* is CoQ-8 [53]. The main polar lipids of strain I5G^T^ are PG, DPG and PE (Table 3, Figure S3). Polar lipid analysis confirms the placement of strain I5G^T^ in the genus *Moraxella*, whose members typically contain PG, CL, MLCL and PE [9,48,54]. In addition, differences in PL1–2, GL and L1–4 profiles were observed between strain I5Gᵀ, *M. nonliquefaciens* CCUG 348ᵀ, *M. oblonga* CCUG 54938ᵀ and *M. nasovis* CCUG 75922^T^, which could serve as reference phenotypic characteristics (Table 3, Figure S3). Therefore, the chemotaxonomic data further support that strain I5G^T^ represents a novel species of the genus *Moraxella*.

**Table 3.** Differential chemotaxonomic characteristics between strain I5G^T^ and the type strains of its closely related species in the genus *Moraxella.*

Characteristic	1	2	3	4	5*	6*	7*	8*
**Saturated**								
C_10:0_	–	4.1	1.9	4.8	1.5		4.7	
C_12:0_	**5.7**	**5.7**	4.4	3.7	3.6	**6.7**	4.4	**6.1**
C_12:0_ 3OH	–	**8.4**	**6.4**	4.9	4.5	3.5	4.0	2.8
C_14:0_	1.3				–	1.7	–	–
C_16:0_	**16.2**	–	**20.0**	**5.6**	4.7	**14.6**	**7.1**	**6.4**
C_17:0_	4.4		1.0		1.7	1.6	–	
C_17:0_iso	–	–	–	1.7	–	–	–	4.0
C_18:0_	**12.7**	3.0	2.7	2.2	1.6	2.6	2.0	2.5
**Unsaturated**								
C_16:1_ ω9c	–	–	–	–	–	–	1.4	1.5
C_17:1_ ω8c	**9.9**	1.2	**7.5**	**12.0**	**24.7**	**11.2**	**9.7**	3.1
C_18:1_ ω9c	**17.2**	**25.9**	**10.2**	**41.0**	**31.4**	**29.5**	**44.4**	**54.8**
C_18:3_ ω6,9,12c	–	–	1.9	1.9	2.0	1.7	3.4	2.4
**Summed features**								
2	–	–	–	–	–	2.5	4.0	4.1
3	**11.6**	**18.2**	**33.3**	**16.8**	**18.6**	**18.3**	**9.7**	**7.8**
**Respiratory quinones**	CoQ-8 (82.5%) CoQ-7 (12.5%) CoQ-9 (5%)	CoQ-8 (96.6%) CoQ-7 (3,4%)	CoQ-8 (96.6%) CoQ-7 (3,4%)	CoQ-8 (97.3 %), CoQ-7 (2.7%)	CoQ-8 (98.5%) CoQ-7 (1.5%)	CoQ-8 (94.7%) CoQ-7 (5.3%)	CoQ-8 (98.3%) CoQ-7 (1.7%)	CoQ-8 (96.7%) CoQ-7 (3.3%)
**Polar lipids** ^†^	DPG, PG, PE, PL1–2, AL1–3, GL, L1–4	DPG, PG, PE, PL1–4, L1–5	DPG, PG, PE, PL1–6, AL1–3, L1–3,	DPG, PG, PE, PL1–5, APL1–2, L1–2	PG, CL, MLCL, HBMP, DG, LPG, PE, Cer-EOS, PC, LPE, Cer-HDS, Cer-NS, DLCL, TG, PA, PS	PG, CL, MLCL, LPG, LPE, DG, PE, PA, DLCL, HBMP, Cer-HDS, DGCC, TG, Cer-EOS	PG, PE, DG, CL, MLCL, HBMP, PC, PA, LPG, LPE, LPC, DLCL, TG	PG, CL, PE, PA, LPG, DG, MLCL, LPE, DLCL, HBMP

* Data from Li et al. [48].

Values are percentage of the total fatty acids and the major fatty acids (>5%) are highlighted in bold type. Fatty acids less than 1.0% in all strains are not shown; –, not detected.

Strains: 1, I5G^T^; 2, *Moraxella nonliquefaciens* CCUG 348^T^; 3, *Moraxella oblonga* CCUG 54938^T^; 4, *Moraxella nasovis* CCUG 75922^T^; 5, *Moraxella bovis* CCUG 2133^T^; 6, *Moraxella nasicaprae* ZY201224^T^; 7, *Moraxella cuniculi* CCUG 2154^T^; 8, *Moraxella caviae* CCUG 355^T^.

^†^DPG, diphosphatidylglycerol; PG, phosphatidylglycerol; PE, phosphatidylethanolamine; CL, cardiolipin; Cer-EOS, ceramide esterified omega-hydroxy fatty acid-sphingosine; Cer-HDS, ceramide hydroxy fatty acid-dihydrosphingosine; DG, diacylglycerol; DGCC, Diacylglyceryl-3-O-carboxyhydroxymethylcholine; DLCL, dilysocardiolipin; HBMP, hemibismonoacylglycerophosphate; LPC, Lysophophatidylcholine; LPE, lysophosphatidylethanolamine; LPG, lysophosphatidylglycerol; MLCL, monolysocardiolipin; PA, phosphatidic acid; PC, phosphatidylcholine; PS, phosphatidylserine; TG, triacylglycerol PL, unidentified phospholipid; AL, unidentified aminolipid; GL, unidentified glycolipid; APL, unidentified aminophospholipid; AGL, unidentified aminoglycolipid; L, unidentified lipid. Numbers following abbreviations indicate different unidentified components detected in the same strain (e.g. PL1–PL4 indicates four distinct unidentified phospholipids).

### Taxonomic conclusion

These phenotypic and genotypic analyses indicate that strain I5G^T^ represents a novel species of the genus *Moraxella*, for which the name *Moraxella salivae* sp. nov. is proposed.

### Description of *Moraxella salivae* sp. nov

*Moraxella salivae* (sa.li'vae. L. gen. *n*. *salivae*, of saliva).

Cells are Gram-negative, rod-shaped and measure approximately 1.5–4.0 µm in length and 0.4–0.6 µm in width. No anaerobic growth is observed. Colonies on Columbia agar after 24 h are 1.0–3.0 mm in diameter, circular, with regular margins, convex, smooth, moist, glistening and grayish-white. No growth occurs on MacConkey agar. The strain grows at 25–37 °C (optimum 37 °C), pH 7.0–9.0 (optimum pH 8.0) and in the presence of 0–1.0% (w/v) NaCl (optimum 0.5% NaCl). No haemolytic activity is detected on 5.0% (v/v) sheep blood agar. Cells are positive for oxidase, esterase (C4), lipase (C8), leucine arylamidase and naphthol-AS-BI-phosphohydrolase activities, but negative for acid phosphatase, catalase, alkaline phosphatase, urease, *β*-galactosidase, arginine dihydrolase, ornithine decarboxylase, gelatinase and tryptophan deaminase. The strain does not assimilate glucose, arabinose, mannose, mannitol, *N*-acetylglucosamine, maltose, gluconic acid, capric acid, adipic acid, malic acid, citric acid and phenylacetic acid. The Voges-Proskauer reaction and glucose fermentation are negative. Nitrate reduction is positive, and hydrogen and indole are not produced. The major fatty acids are C₁₈:₁ ω9c, C₁₆:₀, C₁₈:₀ and Summed Feature 3 (C₁₆:₁ ω7c and/or C₁₆:₁ ω6c). The predominant respiratory quinone is CoQ-8. The main polar lipids are phosphatidylglycerol (PG), diphosphatidylglycerol (DPG) and phosphatidylethanolamine (PE). The genomic DNA G + C content is 37.5 mol%.

The type strain is I5G^T^ (KCTC 18404ᵀ = GDMCC 1.5953ᵀ), isolated from the saliva of a healthy human volunteer in Hebei Province, China.

## Supplementary Material

Supplementary material.pdf Supplementary material.pdf

Culture Collection Certificate.pdf Culture Collection Certificate.pdf

## Data Availability

The GenBank/EMBL/DDBJ/PIR accession numbers for the 16S rRNA gene and draft whole-genome sequence of strain I5G^T^ are PX993265 and JBUXLH000000000.1, respectively.
