# Supplementary material for: Moraxella salivae sp. nov., isolated from the saliva of a healthy human volunteer
Source: J Oral Microbiol. 2026 Sep 23;18(1):2737704. doi: 10.1080/20002297.2026.2737704 (PMC13618153; doi:10.1080/20002297.2026.2737704)
Supplement: Supplementary file 1 [file ZJOM_A_2737704_SM4943.pdf]

# Table S1. Uncultured 16S rRNA gene sequences homologous to strain I5G$^{T}$ retrieved from GenBank

| Accession | Sample source | Alignment coverage | Sequence identity | Sequence length |
|---|---|---|---|---|
| AM420053.1 | subgingival plaque | 99% | 99.37% | 1497 |
| MF578820.1 | periodontal abscess | 97% | 99.71% | 1401 |
| KF080555.1 | skin, volar forearm | 94% | 99.55% | 1357 |
| HM330475.1 | skin, volar forearm | 94% | 99.48% | 1357 |
| HM330474.1 | skin, volar forearm | 94% | 99.48% | 1357 |
| HM330494.1 | skin, volar forearm | 94% | 99.41% | 1357 |
| HM317359.1 | skin, popliteal fossa | 94% | 99.41% | 1357 |
| HM317318.1 | skin, popliteal fossa | 94% | 99.41% | 1357 |
| HM312672.1 | skin, antecubital fossa | 94% | 99.41% | 1357 |
| HM269823.1 | skin, antecubital fossa | 94% | 99.41% | 1357 |
| KF101364.1 | skin, volar forearm | 94% | 99.33% | 1357 |
| JF216181.1 | skin, antecubital fossa | 94% | 99.33% | 1357 |
| JF189147.1 | skin, volar forearm | 94% | 99.33% | 1357 |
| JF171610.1 | skin, volar forearm | 94% | 99.33% | 1357 |
| JF128833.1 | skin, volar forearm | 94% | 99.33% | 1357 |
| JF153384.1 | skin, volar forearm | 94% | 99.26% | 1357 |
| KF101715.1 | skin, volar forearm | 94% | 99.26% | 1357 |
| JF147988.1 | skin, antecubital fossa | 94% | 99.26% | 1357 |
| JF144841.1 | skin, volar forearm | 94% | 99.26% | 1357 |
| JF142905.1 | skin, popliteal fossa | 94% | 99.26% | 1357 |
| KF101038.1 | skin, antecubital fossa | 94% | 99.26% | 1357 |
| JF232149.1 | skin, nare | 94% | 99.26% | 1357 |
| JF135296.1 | skin, volar forearm | 94% | 99.26% | 1357 |
| JF133751.1 | skin, popliteal fossa | 94% | 99.26% | 1357 |
| JF128533.1 | skin, nare | 94% | 99.26% | 1357 |
| JF128425.1 | skin, popliteal fossa | 94% | 99.26% | 1357 |
| JF155228.1 | skin, volar forearm | 94% | 99.26% | 1357 |
| HM256104.1 | skin, nare | 94% | 99.26% | 1357 |
| JF216855.1 | skin, volar forearm | 94% | 99.26% | 1357 |
| JF144921.1 | skin, volar forearm | 93% | 99.33% | 1357 |
| KF100520.1 | skin, antecubital fossa | 94% | 99.18% | 1357 |
| KF098335.1 | skin, volar forearm | 94% | 99.18% | 1357 |
| JF218190.1 | skin, volar forearm | 94% | 99.18% | 1356 |
| JF203409.1 | skin, nare | 94% | 99.18% | 1357 |
| JF154677.1 | skin, antecubital fossa | 94% | 99.18% | 1357 |
| JF154637.1 | skin, antecubital fossa | 94% | 99.18% | 1357 |
| JF149724.1 | skin, volar forearm | 94% | 99.18% | 1357 |
| JF147842.1 | skin, antecubital fossa | 94% | 99.18% | 1357 |
| JF147025.1 | skin, popliteal fossa | 94% | 99.18% | 1357 |
| JF144945.1 | skin, volar forearm | 94% | 99.18% | 1356 |
| JF141736.1 | skin, volar forearm | 94% | 99.18% | 1357 |
| JF140165.1 | skin, popliteal fossa | 94% | 99.18% | 1357 |
| JF203702.1 | skin, volar forearm | 94% | 99.18% | 1356 |
| JF140111.1 | skin, popliteal fossa | 94% | 99.18% | 1357 |
| JF139496.1 | skin, volar forearm | 94% | 99.18% | 1357 |
| JF135990.1 | skin, popliteal fossa | 94% | 99.18% | 1357 |
| KF098510.1 | skin, antecubital fossa | 94% | 99.11% | 1357 |
| JF218312.1 | skin, volar forearm | 94% | 99.11% | 1356 |
| JF197304.1 | skin, volar forearm | 94% | 99.11% | 1357 |
| JF172276.1 | skin, popliteal fossa | 94% | 99.11% | 1357 |
| JF151840.1 | skin, volar forearm | 94% | 99.11% | 1357 |
| JF149845.1 | skin, volar forearm | 94% | 99.11% | 1357 |
| JF148817.1 | skin, popliteal fossa | 94% | 99.11% | 1357 |
| JF148522.1 | skin, volar forearm | 94% | 99.11% | 1357 |
| JF148364.1 | skin, volar forearm | 94% | 99.11% | 1357 |
| JF149157.1 | skin, antecubital fossa | 94% | 99.11% | 1357 |
| KF100560.1 | skin, volar forearm | 94% | 99.11% | 1357 |
| JF140261.1 | skin, popliteal fossa | 94% | 99.11% | 1357 |
| JF133046.1 | skin, antecubital fossa | 94% | 99.11% | 1357 |
| HM252615.1 | skin, antecubital fossa | 94% | 99.11% | 1357 |
| JF148689.1 | skin, popliteal fossa | 94% | 99.11% | 1357 |
| JF135244.1 | skin, volar forearm | 94% | 99.04% | 1358 |
| HM255969.1 | skin, nare | 94% | 99.04% | 1359 |
| HM252542.1 | skin, antecubital fossa | 93% | 99.18% | 1357 |
| KF100543.1 | skin, antecubital fossa | 94% | 99.04% | 1357 |
| JF203750.1 | skin, volar forearm | 94% | 99.04% | 1356 |
| JF188558.1 | skin, volar forearm | 94% | 99.04% | 1357 |
| JF170017.1 | skin, volar forearm | 94% | 99.04% | 1357 |
| JF151375.1 | skin, volar forearm | 94% | 99.04% | 1357 |
| JF148607.1 | skin, popliteal fossa | 94% | 99.04% | 1357 |
| JF146913.1 | skin, popliteal fossa | 94% | 99.04% | 1357 |
| JF142801.1 | skin, volar forearm | 94% | 99.04% | 1357 |
| JF142728.1 | skin, antecubital fossa | 94% | 99.04% | 1357 |
| JF142386.1 | skin, popliteal fossa | 94% | 99.04% | 1357 |

Table S2. Genomic metadata of *Moraxella* type strains used for genomic comparisons

| Name | Accession | Assembly level | Data source | Download date |
|---|---|---|---|---|
| *Moraxella caprae* DSM 19149$^T$ | GCF_900453285.1 | Scaffold | NCBI Assembly | 2025-06-08 |
| *Moraxella bovoculi* 237$^T$ | GCF_000696305.2 | Contig | NCBI Assembly | 2025-06-08 |
| *Moraxella osloensis* CCUG 350$^T$ | GCF_001553955.1 | Complete | NCBI Assembly | 2025-06-08 |
| *Moraxella lacunata* NBRC 102154$^T$ | GCF_001591245.1 | Contig | NCBI Assembly | 2025-06-08 |
| *Moraxella oblonga* NBRC102422$^T$ | GCF_001598275.1 | Contig | NCBI Assembly | 2025-06-08 |
| *Moraxella catarrhalis* CCUG 353$^T$ | GCF_001632285.1 | Scaffold | NCBI Assembly | 2025-06-08 |
| *Moraxella ovis* 199/55$^T$ | GCF_001636015.1 | Complete | NCBI Assembly | 2025-06-08 |
| *Moraxella lincolnii* CCUG 9405$^T$ | GCF_002014765.1 | Scaffold | NCBI Assembly | 2025-06-08 |
| *Moraxella pluranimalium* CCUG 54913$^T$ | GCF_002014825.1 | Scaffold | NCBI Assembly | 2025-06-08 |
| *Moraxella porci* DSM 25326$^T$ | GCF_002014855.1 | Scaffold | NCBI Assembly | 2025-06-08 |
| *Moraxella canis* CCUG 8415A$^T$ | GCF_002014965.1 | Scaffold | NCBI Assembly | 2025-06-08 |
| *Moraxella bovis* CCUG 2133$^T$ | GCF_002014975.1 | Scaffold | NCBI Assembly | 2025-06-08 |
| *Moraxella equi* CCUG 4950$^T$ | GCF_002027555.1 | Scaffold | NCBI Assembly | 2025-06-08 |
| *Moraxella nasovis* ZY201115$^T$ | GCF_022701215.1 | Complete | NCBI Assembly | 2025-06-08 |
| *Moraxella nasicaprae* ZY201224$^T$ | GCF_025643275.1 | Complete | NCBI Assembly | 2025-06-08 |
| *Moraxella cuniculi* DSM 21768$^T$ | GCF_900156515.1 | Contig | NCBI Assembly | 2025-06-08 |
| *Moraxella oculi* Tifton1$^T$ | GCF_023336735.1 | Contig | NCBI Assembly | 2025-06-08 |
| *Moraxella haemolytica* ZY171148$^T$ | GCF_030177935.1 | Complete | NCBI Assembly | 2025-06-08 |
| *Moraxella nonliquefaciens* CCUG 348$^T$ | GCF_001679005.1 | Scaffold | NCBI Assembly | 2025-06-08 |
| *Moraxella caviae* CCUG 355$^T$ | GCF_002014985.1 | Scaffold | NCBI Assembly | 2025-06-08 |

# Table S3. BLASTp matching information against the VFDB virulence factor database for strain I5G$^T$

| SeqID | Identity (%) | Query coverage (%) | Accession | Annotation |
|---|---|---|---|---|
| ctg_00990 | 81.395 | 96 | VFG046475(gb\|WP_013922406) | (tufA) elongation factor Tu [EF-Tu (VF0460) - Adherence (VFC0001)] [*Francisella* sp. TX077308] |
| ctg_01009 | 63.077 | 96 | VFG013197(gb\|WP_011608705) | (hemB) porphobilinogen synthase [Heme biosynthesis (VF0758) - Nutritional/Metabolic factor (VFC0272)] [*Haemophilus somnus* 2336] |
| ctg_01043 | 65.919 | 98 | VFG044201(gb\|WP_011267793) | (PSYR_RS13390) diaminobutyrate--2-oxoglutarate transaminase [Achromobactin biosynthesis and transport (VF0922) - Nutritional/Metabolic factor (VFC0272)] [*Pseudomonas syringae* pv. *syringae* B728a] |
| ctg_01066 | 72.328 | 96 | VFG001855(gb\|WP_197535493) | (htpB) Hsp60, 60K heat shock protein HtpB [Hsp60 (VF0159) - Adherence (VFC0001)] [*Legionella pneumophila* subsp. *pneumophila* str. Philadelphia 1] |
| ctg_01208 | 81.395 | 96 | VFG046475(gb\|WP_013922406) | (tufA) elongation factor Tu [EF-Tu (VF0460) - Adherence (VFC0001)] [*Francisella* sp. TX077308] |
| ctg_01214 | 62.891 | 96 | VFG037798(gb\|WP_000064877) | (lpxA) acyl-ACP--UDP-N-acetylglucosamine O-acyltransferase [LPS (VF0466) - Immune modulation (VFC0258)] [*Acinetobacter baumannii* D1279779] |
| ctg_01237 | 62.678 | 98 | VFG049194(gb\|WP_004150973) | (clpV) ATP-dependent chaperone ClpB [T6SS-II (VF0783) - Effector delivery system (VFC0086)] [*Klebsiella pneumoniae* JM45] |
| ctg_01333 | 63.538 | 96 | VFG013470(gb\|WP_011609100) | (kdsA) 2-dehydro-3-deoxyphosphooctonate aldolase [LOS (VF0044) - Immune modulation (VFC0258)] [*Haemophilus somnus* 129PT] |

| SeqID | Identity (%) | Query coverage (%) | Accession | Annotation |
|---|---|---|---|---|
| ctg_00467 | 60.615 | 90 | VFG006199(gb\|WP_010980842) | (pilT2) twitching motility protein PilT [Type IV pili (VF0075) - Adherence (VFC0001)] [*Neisseria meningitidis* MC58] |
| ctg_00530 | 68.382 | 99 | VFG050470(gb\|WP_000279216.1) | (pilC) type II secretion system F family protein [TFP (VF1334) - Adherence (VFC0001)] [*Acinetobacter baumannii* ACICU] |
| ctg_00550 | 85.169 | 98 | VFG038216(gb\|WP_000076440) | (bfmR) biofilm-controlling response regulator [BfmRS (VF0463) - Regulation (VFC0301)] [*Acinetobacter baumannii* 1656-2] |
| ctg_01698 | 61.894 | 95 | VFG043580(gb\|WP_003662682) | (MCR_RS07635) OmpA family protein [TaaP (VF1224) - Adherence (VFC0001)] [*Moraxella catarrhalis* RH4] |
| ctg_00878 | 62.23 | 97 | VFG012585(gb\|WP_001101723) | (sitC) iron/manganese ABC transporter permease subunit SitC [Iron/manganese transport (VF1116) - Nutritional/Metabolic factor (VFC0272)] [*Escherichia coli* APEC O1] |
| ctg_00902 | 66.047 | 100 | VFG036719(gb\|WP_002243173) | (ctrD) capsule polysaccharide export ATP-binding protein CtrD [Capsule (VF0079) - Immune modulation (VFC0258)] [*Neisseria meningitidis* H44/76] |
| ctg_01767 | 61.502 | 100 | VFG013618(gb\|WP_011609384) | (hemL) glutamate-1-semialdehyde 2,1-aminomutase [Heme biosynthesis (VF0758) - Nutritional/Metabolic factor (VFC0272)] [*Haemophilus somnus* 129PT] |

| SeqID | Identity (%) | Query coverage (%) | Accession | Annotation |
|---|---|---|---|---|
| ctg_01837 | 66.903 | 99 | VFG050461(gb\|WP_001274989.1) | (pilB) type IV-A pilus assembly ATPase PilB [TFP (VF1334) - Adherence (VFC0001)] [*Acinetobacter baumannii* BJAB0715] |
| ctg_01839 | 60.638 | 86 | VFG000077(gb\|NP_465991) | (clpP) ATP-dependent Clp protease proteolytic subunit [ClpP (VF0074) - Stress survival (VFC0282)] [*Listeria monocytogenes* EGD-e] |
| ctg_01859 | 90.78 | 100 | VFG042380(gb\|ABQ43328) | (ABQ43328) MhaB2 [MhaB2-MhaC (VF1277) - Effector delivery system (VFC0086)] [*Moraxella catarrhalis* str. O35E] |
| ctg_01468 | 69.459 | 98 | VFG050398(gb\|WP_000347036.1) | (pilU) PilT/PilU family type 4a pilus ATPase [TFP (VF1334) - Adherence (VFC0001)] [*Acinetobacter baumannii* AB0057] |
| ctg_01469 | 81.341 | 99 | VFG050386(gb\|WP_000355489.1) | (pilT) type IV pilus twitching motility protein PilT [TFP (VF1334) - Adherence (VFC0001)] [*Acinetobacter baumannii* ACICU] |
| ctg_00261 | 65.272 | 99 | VFG038840(gb\|WP_005300916) | (flmH) short chain dehydrogenase/reductase family oxidoreductase [Polar flagella (VF0473) - Motility (VFC0204)] [*Aeromonas hydrophila* ML09-119] |
| ctg_00298 | 63.368 | 89 | VFG043573(gb\|NP_219906) | (dnaK) chaperone protein DnaK [Adherence; porin (VF0713) - Adherence (VFC0001)] [*Chlamydia trachomatis* D/UW-3/CX] |
| ctg_00315 | 66.387 | 99 | VFG050640(gb\|WP_000101096.1) | (pilH) response regulator [TFP (VF1334) - Adherence (VFC0001)] [*Acinetobacter baumannii* ACICU] |
| ctg_00316 | 75.833 | 97 | VFG050625(gb\|WP_000389061.1) | (pilG) twitching motility response regulator PilG [TFP (VF1334) - Adherence (VFC0001)] [*Acinetobacter baumannii* ACICU] |
| ctg_00047 | 63.543 | 98 | VFG050772(gb\|WP_001020922.1) | (gspE2) putative secretion pathway ATPase [T2SS (VF1335) - Effector delivery system (VFC0086)] [*Acinetobacter baumannii* ACICU] |
| ctg_00078 | 62.896 | 98 | VFG046604(gb\|WP_012280804) | (rpe) ribulose-phosphate 3-epimerase [Capsule (VF0543) - Immune modulation (VFC0258)] [*Francisella philomiragia* subsp. philomiragia ATCC 25017] |

Table S4. Sequence similarities of 16S rRNA gene sequences between strain I5G$^T$ and the type strains of its closely related species in the genus *Moraxella*

| Name | Strain | Accession | Similarity(%) |
|---|---|---|---|
| *Moraxella oblonga* | IAM 14971$^T$ | AB087260 | 96.17 |
| *Moraxella equi* | CCUG 4950$^T$ | MXAP01000027 | 96.09 |
| *Moraxella lacunata* | NBRC 102154$^T$ | BCUK01000202 | 95.95 |
| *Moraxella nasibovis* | ZY190618$^T$ | OL841892 | 95.95 |
| *Moraxella bovoculi* | 237$^T$ | AOMT01000014 | 95.88 |
| *Moraxella nasicaprae* | ZY201224$^T$ | GCA_025643275.1_1 | 95.73 |
| *Moraxella bovis* | CCUG 2133$^T$ | MUXV01000051 | 95.66 |
| *Moraxella oculi* | Tifton1$^T$ | ON062946 | 95.66 |
| *Moraxella caprae* | DSM 19149$^T$ | AUGF01000070 | 95.54 |
| *Moraxella nonliquefaciens* | NCTC 7784$^T$ | AF005172 | 95.38 |
| *Moraxella cuniculi* | DSM 21768$^T$ | jgi.1011111 | 95.38 |
| *Moraxella haemolytica* | ZY171148$^T$ | CP089974 | 95.17 |
| *Moraxella nasovis* | ZY201115$^T$ | OL841893 | 95.10 |
| *Moraxella ovis* | 199/55$^T$ | CP011158 | 95.09 |
| *Moraxella porci* | CCUG 54912$^T$ | MUYV01000016 | 95.09 |
| *Moraxella canis* | CCUG 8415A$^T$ | MUXT01000012 | 94.95 |
| *Moraxella pluranimalium* | CCUG 54913$^T$ | MUYU01000024 | 94.95 |
| *Moraxella caviae* | CCUG 355$^T$ | MUXU01000060 | 94.88 |
| *Moraxella catarrhalis* | CCUG 353$^T$ | LWAH01000018 | 94.81 |

Table S5. OrthoANI values between strain I5G$^T$ and the type strains of its closely related species in the genus *Moraxella*

| Query genome | Referencegenome | ANI |
|---|---|---|
| I5G$^T$ | *Moraxella caprae* DSM 19149$^T$ | 85% |
| I5G$^T$ | *Moraxella bovoculi* 237$^T$ | 85% |
| I5G$^T$ | *Moraxella osloensis* CCUG 350$^T$ | 84% |
| I5G$^T$ | *Moraxella lacunata* NBRC 102154$^T$ | 85% |
| I5G$^T$ | *Moraxella oblonga* NBRC102422$^T$ | 84% |
| I5G$^T$ | *Moraxella catarrhalis* CCUG 353$^T$ | 85% |
| I5G$^T$ | *Moraxella ovis* 199/55$^T$ | 85% |
| I5G$^T$ | *Moraxella lincolnii* CCUG 9405$^T$ | 85% |
| I5G$^T$ | *Moraxella pluranimalium* CCUG 54913$^T$ | 84% |
| I5G$^T$ | *Moraxella porci* DSM 25326$^T$ | 85% |
| I5G$^T$ | *Moraxella canis* CCUG 8415A$^T$ | 86% |
| I5G$^T$ | *Moraxella bovis* CCUG 2133$^T$ | 86% |
| I5G$^T$ | *Moraxella equi* CCUG 4950$^T$ | 85% |
| I5G$^T$ | *Moraxella nasovis* ZY201115$^T$ | 85% |
| I5G$^T$ | *Moraxella nasicaprae* ZY201224$^T$ | 85% |
| I5G$^T$ | *Moraxella cuniculi* DSM 21768$^T$ | 85% |
| I5G$^T$ | *Moraxella oculi* Tifton1$^T$ | 71% |
| I5G$^T$ | *Moraxella haemolytica* ZY171148$^T$ | 71% |
| I5G$^T$ | *Moraxella nonliquefaciens* CCUG 348$^T$ | 71% |
| I5G$^T$ | *Moraxella caviae* CCUG 355$^T$ | 71% |

Table S6. dDDH values between strain I5G$^T$ and the type strains of its closely related species in the genus *Moraxella*

| Query genome | Referencegenome | DDH |
| --- | --- | --- |
| I5G$^T$ | *Moraxella caprae* DSM19149$^T$ | 22.3 |
| I5G$^T$ | *Moraxella bovoculi* 237$^T$ | 23.5 |
| I5G$^T$ | *Moraxella osloensis* CCUG350$^T$ | 23.5 |
| I5G$^T$ | *Moraxella lacunata* NBRC102154$^T$ | 21.5 |
| I5G$^T$ | *Moraxella oblonga* NBRC102422$^T$ | 20.9 |
| I5G$^T$ | *Moraxella catarrhalis* CCUG353$^T$ | 22.6 |
| I5G$^T$ | *Moraxella ovis* 199/55$^T$ | 24 |
| I5G$^T$ | *Moraxella lincolnii* CCUG9405$^T$ | 21.8 |
| I5G$^T$ | *Moraxella pluranimalium* CCUG54913$^T$ | 23.9 |
| I5G$^T$ | *Moraxella porci* DSM25326$^T$ | 22.5 |
| I5G$^T$ | *Moraxella canis* CCUG8415A$^T$ | 23.2 |
| I5G$^T$ | *Moraxella bovis* CCUG2133$^T$ | 22.5 |
| I5G$^T$ | *Moraxella equi* CCUG4950$^T$ | 21.9 |
| I5G$^T$ | *Moraxella nasovis* ZY201115$^T$ | 24.1 |
| I5G$^T$ | *Moraxella nasicaprae* ZY201224$^T$ | 24.8 |
| I5G$^T$ | *Moraxella cuniculi* DSM21768$^T$ | 24.3 |
| I5G$^T$ | *Moraxella oculi* Tifton1$^T$ | 22,3 |
| I5G$^T$ | *Moraxella haemolytica* ZY171148$^T$ | 24.2 |
| I5G$^T$ | *Moraxella nonliquefaciens* CCUG 348$^T$ | 22.0 |
| I5G$^T$ | *Moraxella caviae* CCUG 355$^T$ | 22.0 |

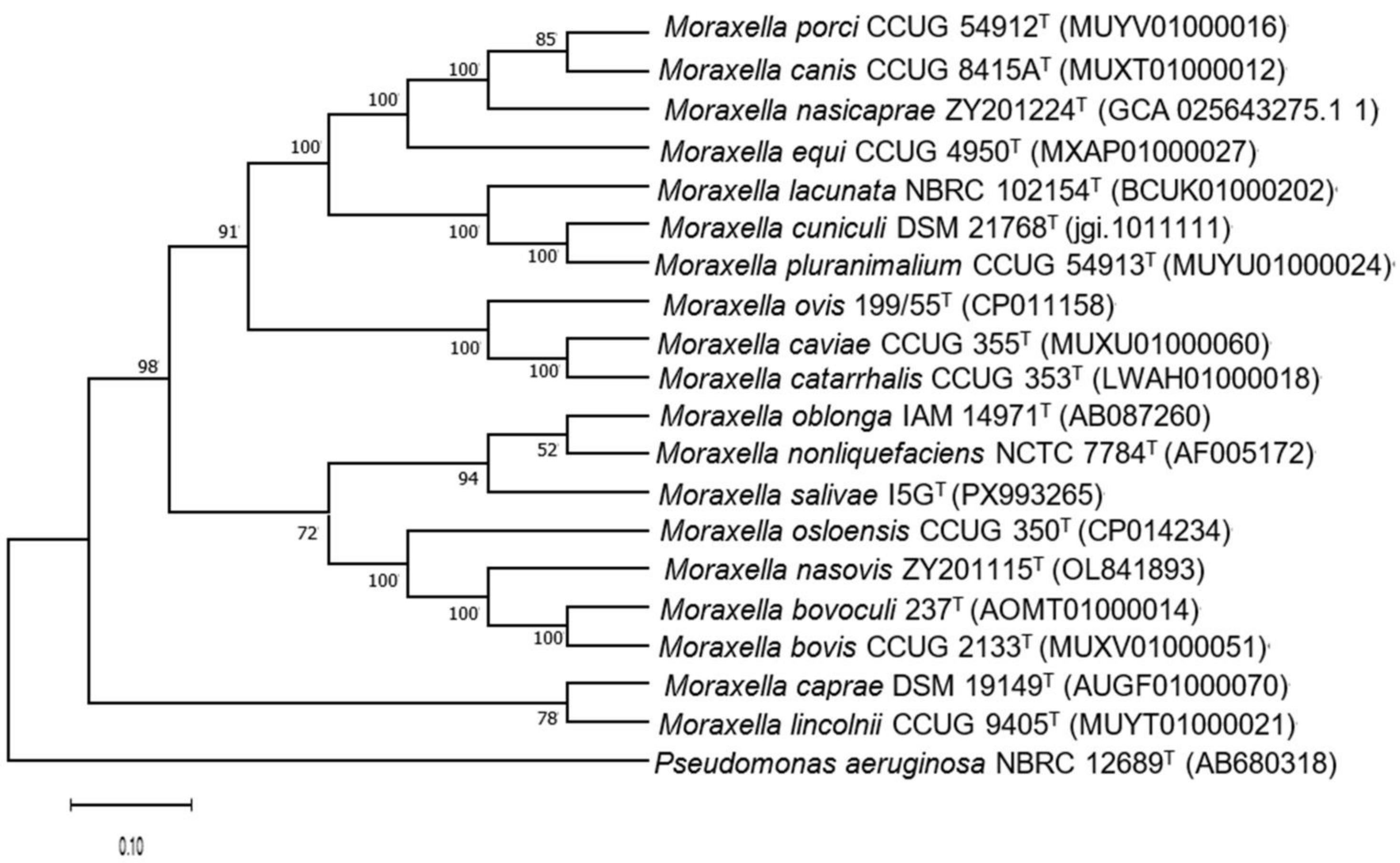


Figure S1. Minimum-evolution phylogenetic tree based on 16S rRNA gene sequences, showing the position between strain I5G$^T$ and type strains in the genus *Moraxella.* Bootstrap values above 50% are shown at branch points. *Pseudomonas aeruginosa* NBRC 12689T was used as an outgroup. Bar, 0.1 substitutions per nucleotide position.

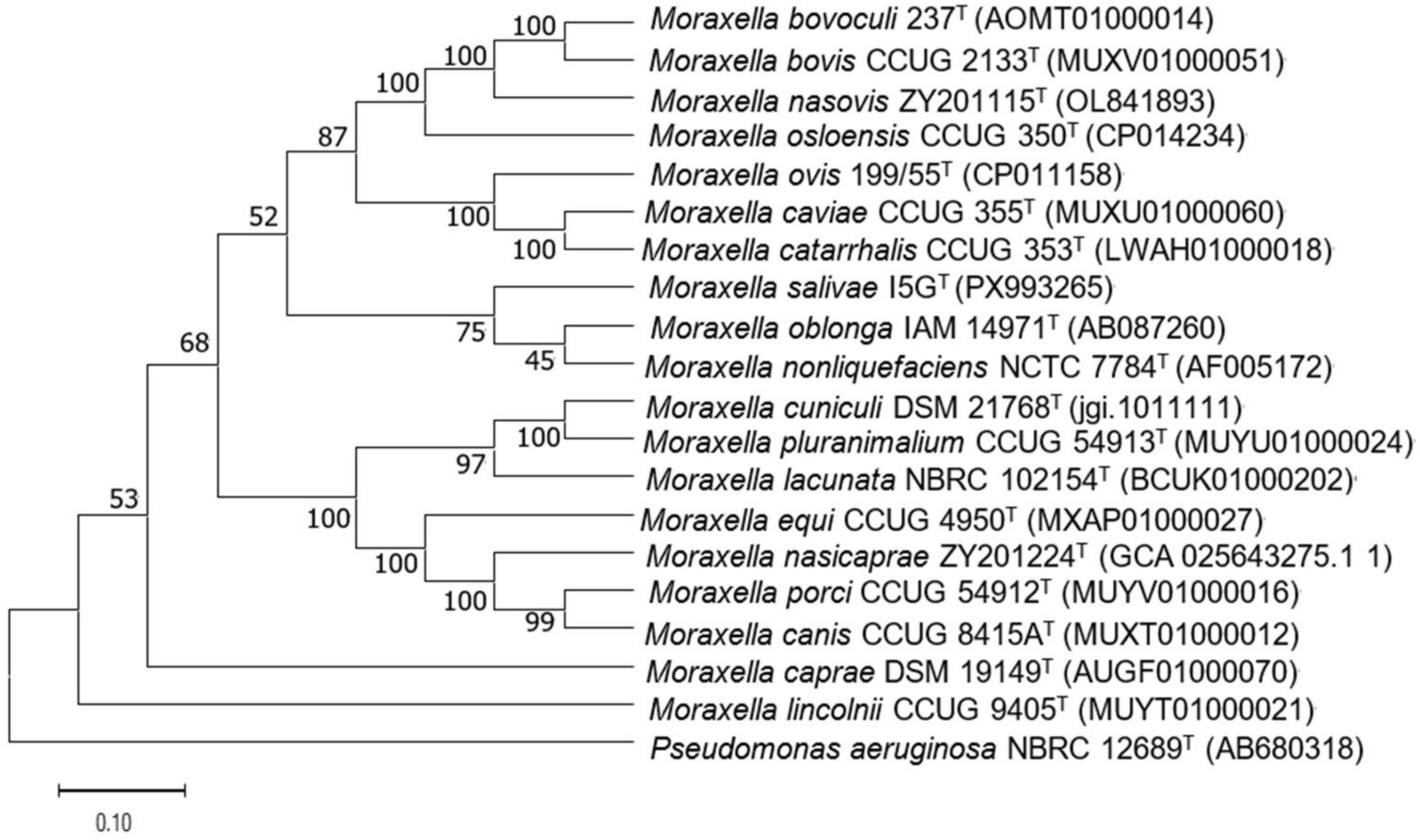


Figure S2. Maximum-likelihood phylogenetic tree based on 16S rRNA gene sequences, showing the position between strain I5G$^{T}$ and type strains in the genus *Moraxella*. Bootstrap values above 50% are shown at branch points. *Pseudomonas aeruginosa* NBRC 12689T was used as an outgroup.

1

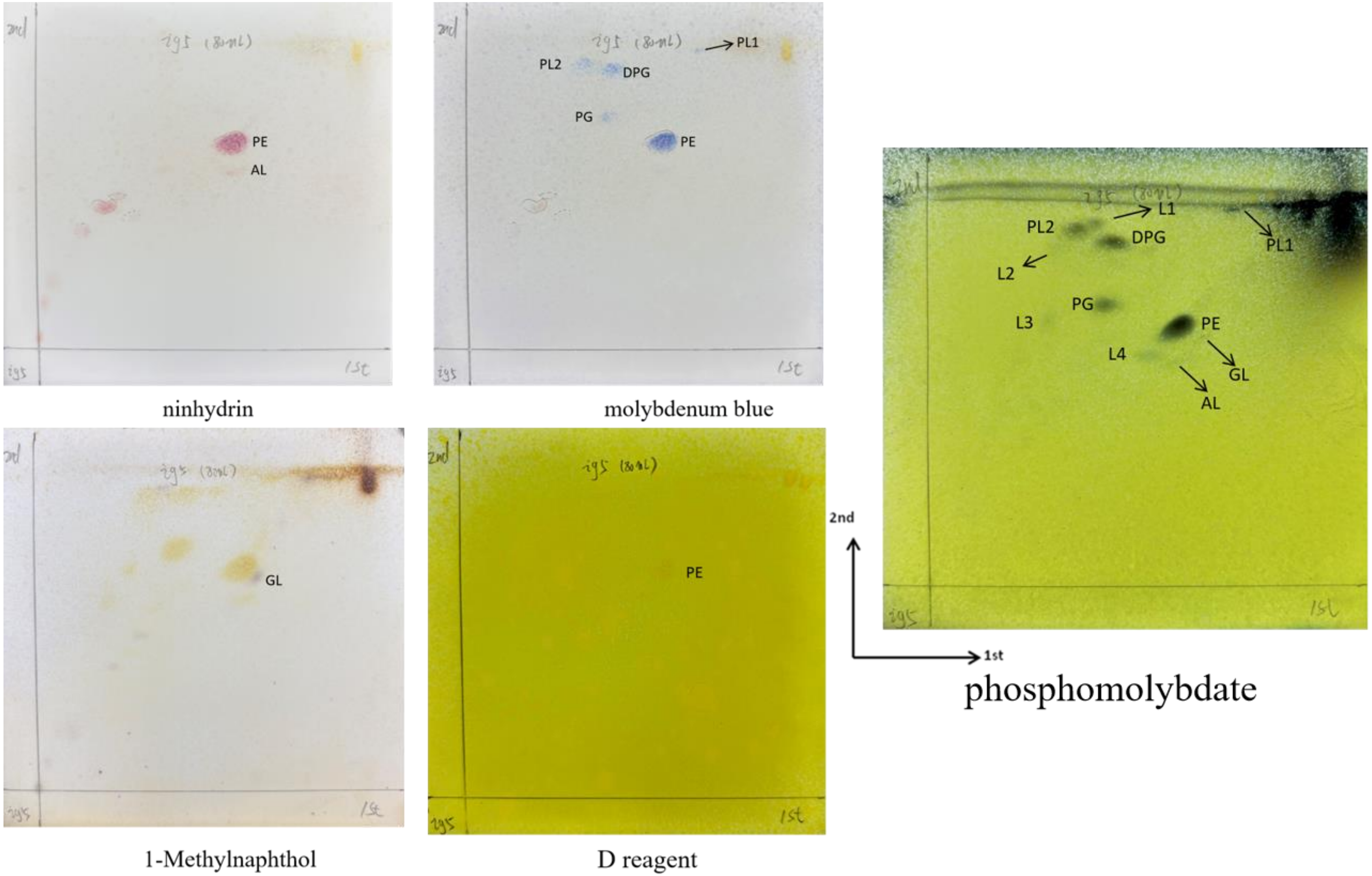
PE
AL
ninhydrin
PL2
DPG
PL1
PG
PE
molybdenum blue
GL
1-Methylnaphthol
PE
D reagent
L1
PL2
DPG
PL1
L2
PG
L3
PE
L4
GL
AL
2nd
1st
phosphomolybdate

2

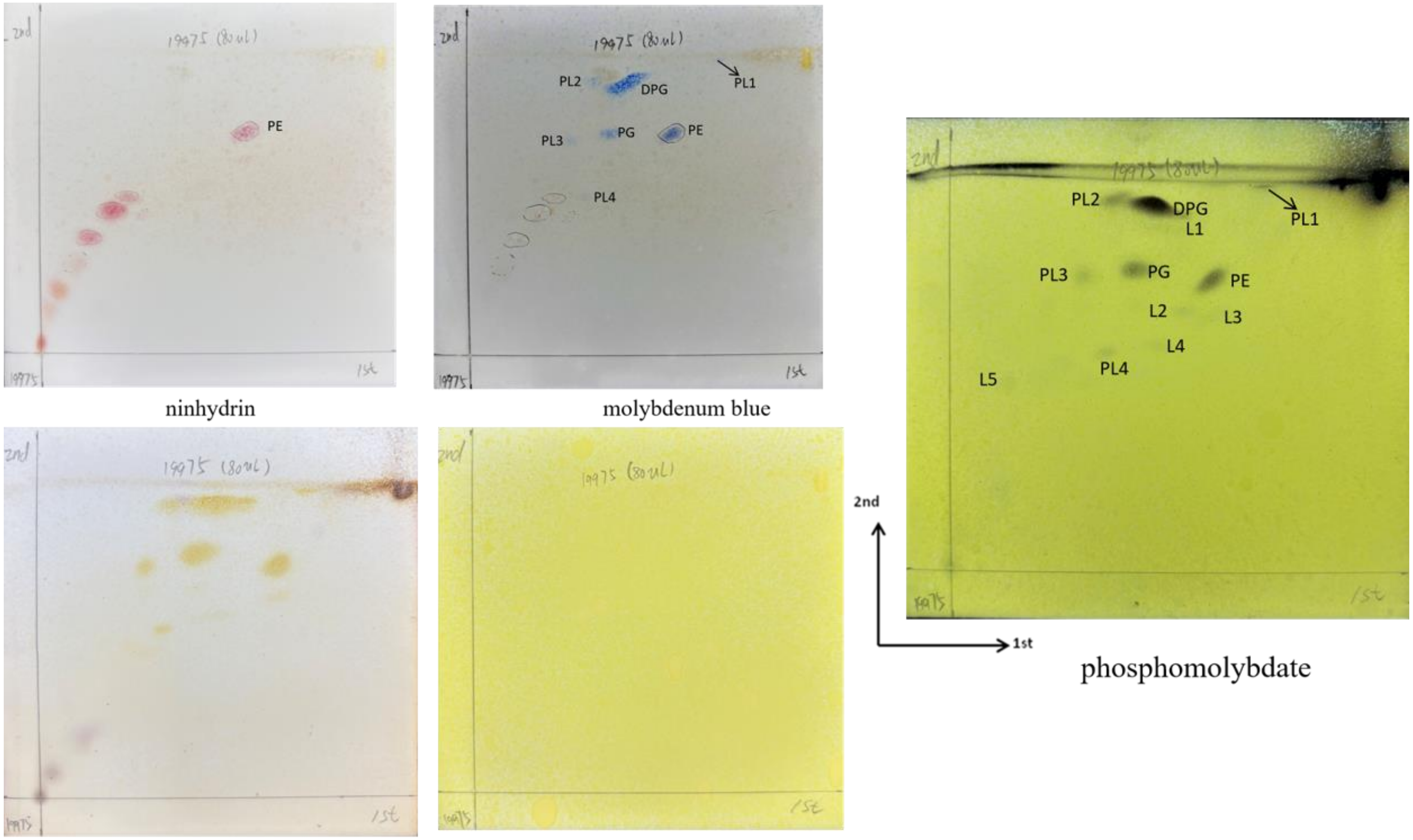


ninhydrin

molybdenum blue

1-Methylnaphthol

D reagent

phosphomolybdate

3

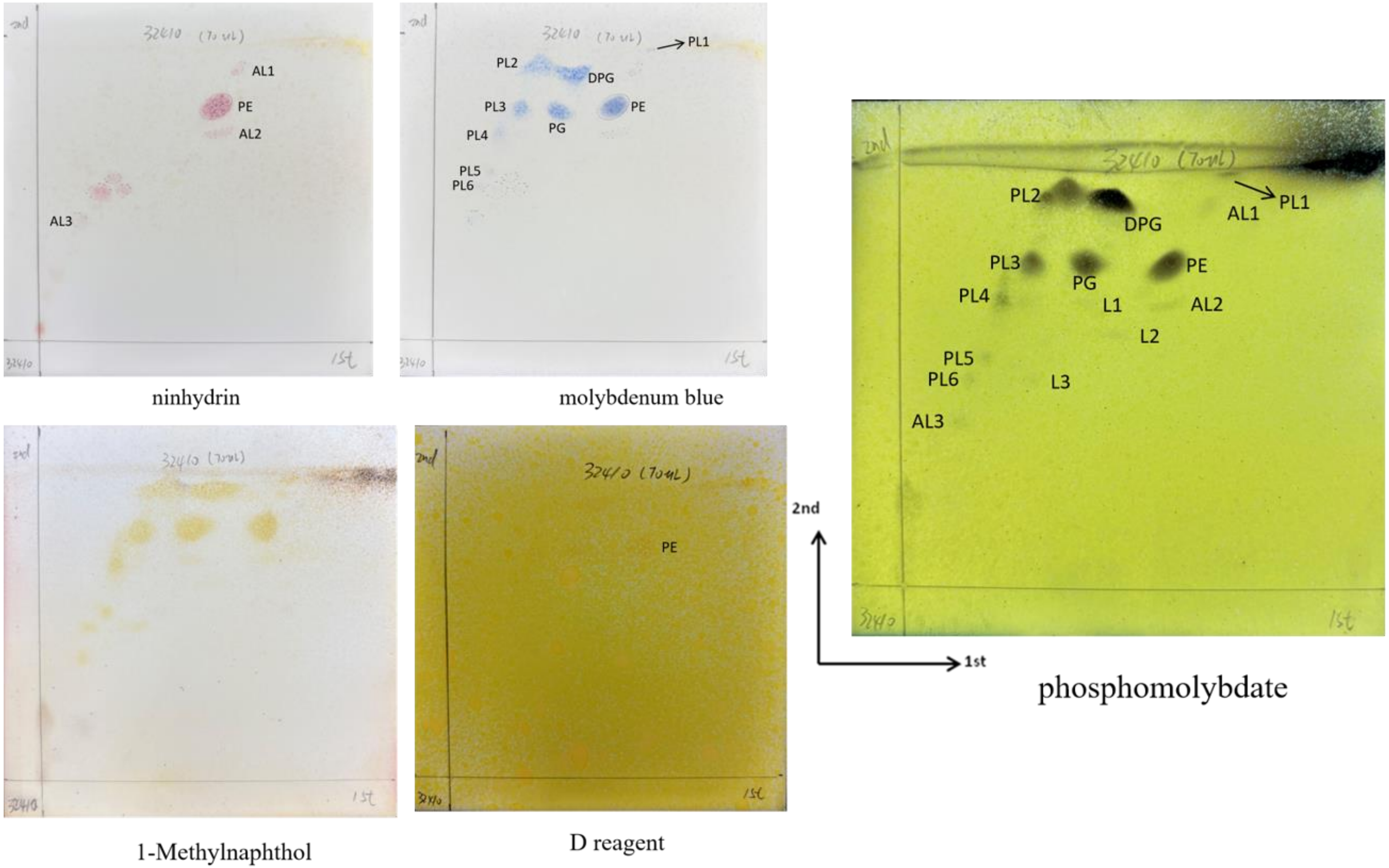

32410 (70 uL)
2nd
1st
AL1
PE
AL2
AL3
ninhydrin
PL1
PL2
DPG
PL3
PG
PL4
PL5
PL6
molybdenum blue
1-Methylnaphthol
PE
D reagent
PL2
DPG
AL1
PL1
PL3
PG
PE
PL4
L1
AL2
L2
PL5
PL6
L3
AL3
phosphomolybdate

4

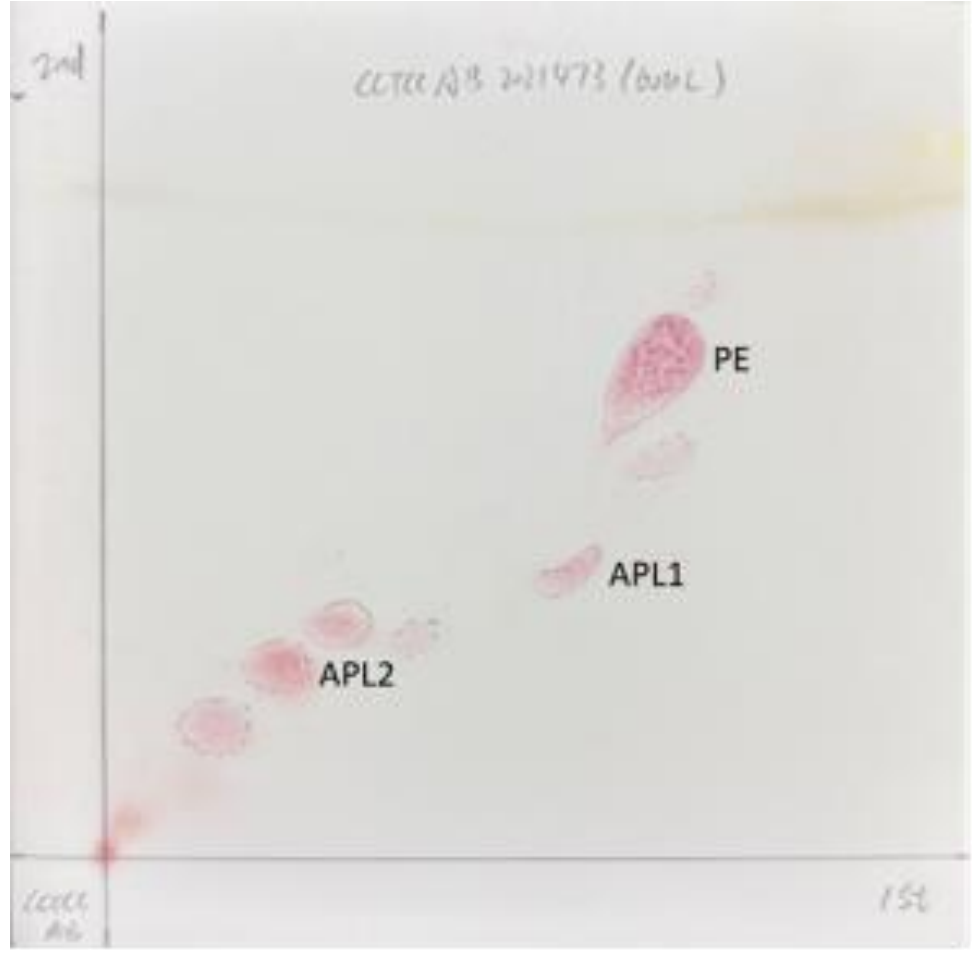


ninhydrin

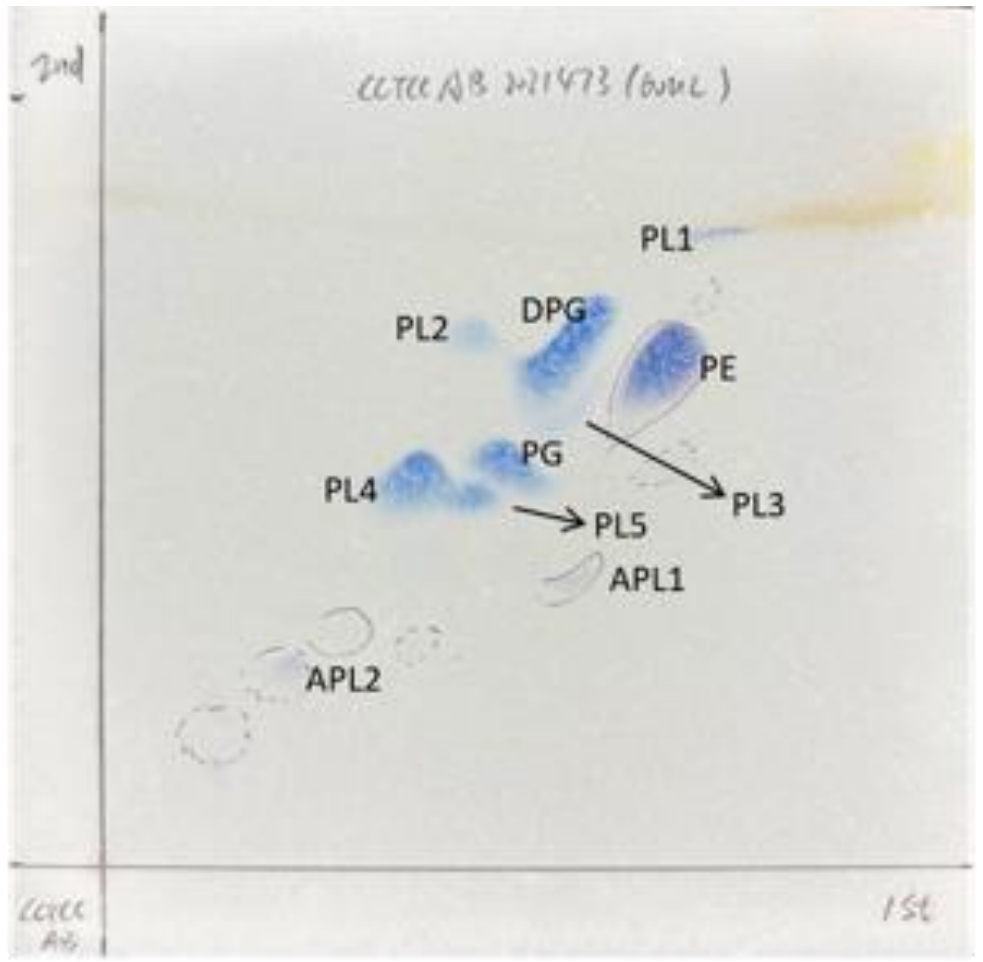


molybdenum blue

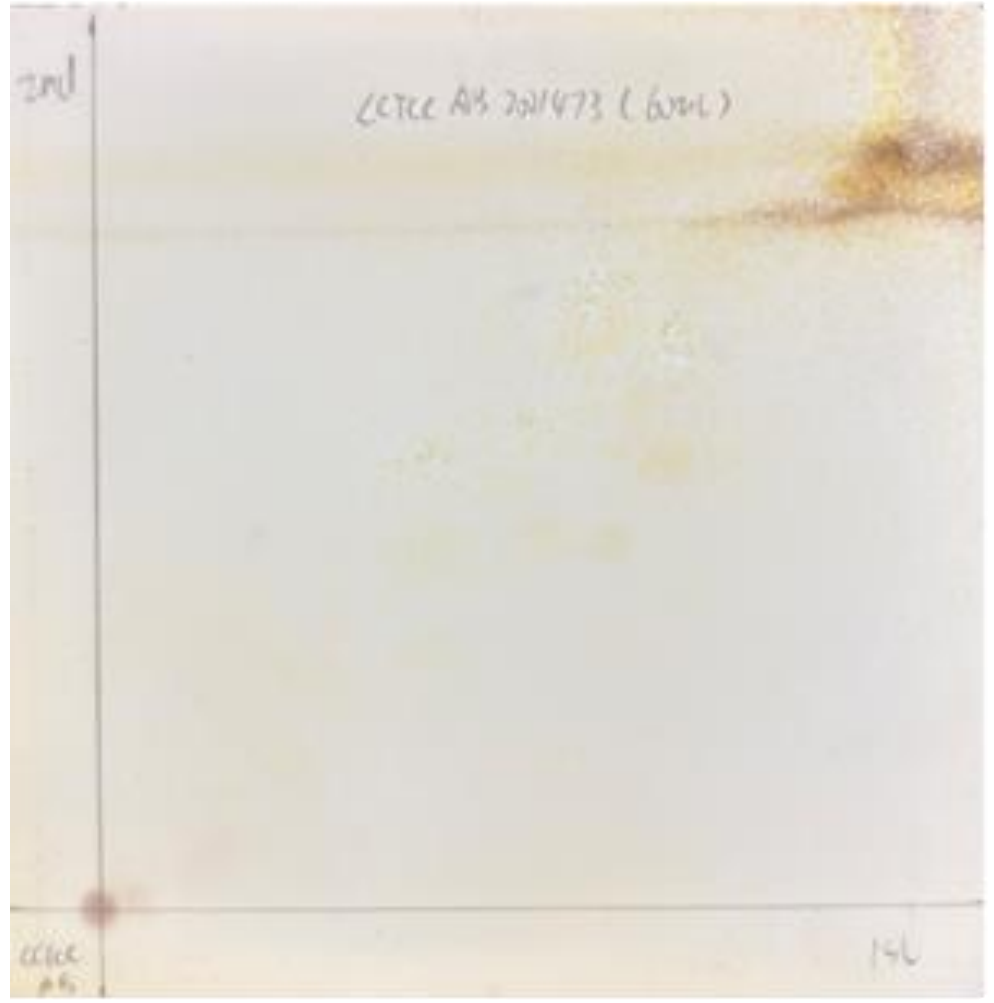

1-Methylnaphthol

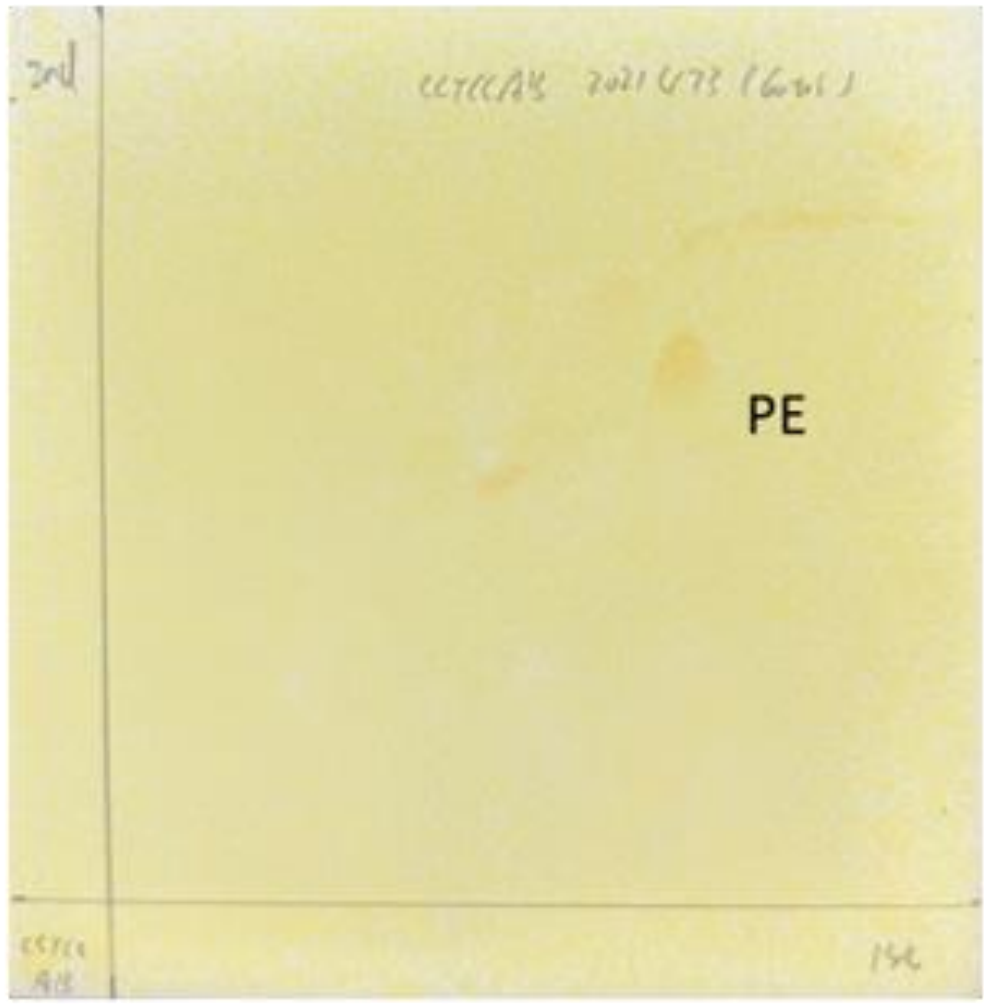


D reagent

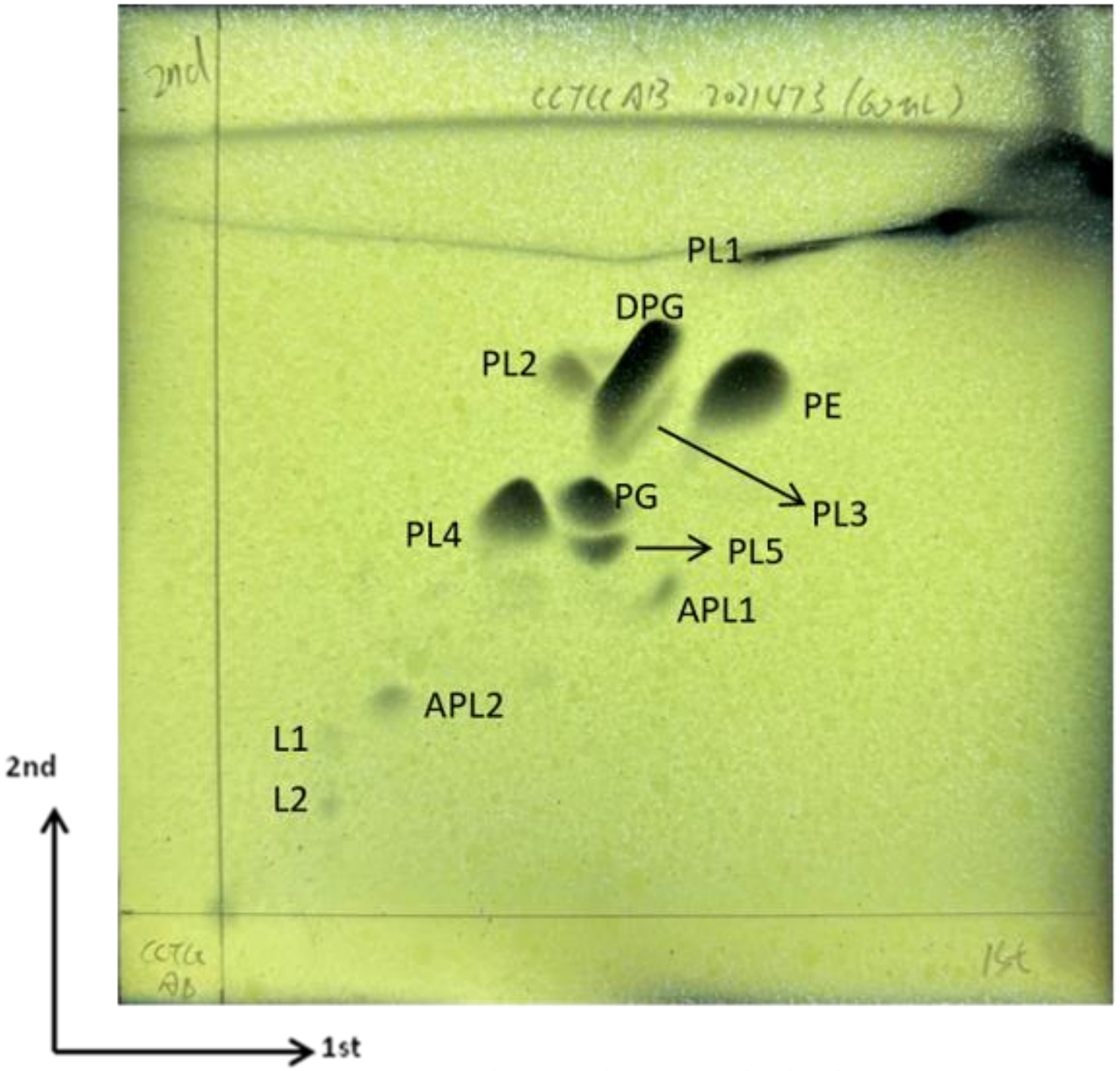


phosphomolybdate

Figure S3. Two-dimensional thin-layer chromatography of polar lipids of strain I5G$^T$ and type strains of its closely related species in the genus *Moraxella*.

Strains: 1, I5G$^T$; 2, *Moraxella nonliquefaciens* CCUG 348$^T$; 3, *Moraxella oblonga* CCUG 54938$^T$; 4, *Moraxella nasovis* CCUG 75922$^T$

DPG, diphosphatidylglycerol; PG, phosphatidylglycerol; PE, phosphatidylethanolamine; PL, unidentified phospholipid; AL, unidentified aminolipid; GL, unidentified glycolipid; APL, unidentified aminophospholipid; AGL, unidentified aminoglycolipid; L, unidentified lipid. Numbers following abbreviations indicate different unidentified components detected in the same strain (e.g., PL1–PL4 indicates four distinct unidentified phospholipids)
