# Supplementary material for: Moraxella salivae sp. nov., isolated from the saliva of a healthy human volunteer
Source: J Oral Microbiol. 2026 Sep 23;18(1):2737704. doi: 10.1080/20002297.2026.2737704 (PMC13618153; doi:10.1080/20002297.2026.2737704)
Supplement: Supplementary file 2 [file ZJOM_A_2737704_SM4944.pdf]

Korean Collection for Type Cultures (KCTC)
Korea Research Institute of Bioscience and Biotechnology (KRIBB)
181 Ipsin-gil, Jeongeup-si, Jeonbuk 56212, South Korea
Tel: +82-63-570-5602, FAX: +82-63-570-5609
E-mail: deposit@kribb.re.kr

Certificate of Deposit

Ref.: 20251100 Date of issue: NOV 10, 2025

Taxonomic designation	:	*Moraxella salivae*
Accession number	:	KCTC 18404
Depositor(s)	:	Zhe Ma
Strain code by the depositor(s)	:	I5G

The above microorganism has been successfully deposited into the general collection of microorganism of the Korean Collection for Type Cultures (KCTC) and confirmed the identity of the microorganism under this KCTC number.
This microorganism will be available without restrictions for research and academic purposes in the publicly accessible section of the KCTC. It will be included in published and online catalogues after publication of this number by the authors.

SongGun Kim

Curator of Bacteria
Song-Gun Kim Ph.D.

Telephone: +82-63-570-5600
Fax: +82-63-570-5609
E-mail: sgkim@kribb.re.kr
web: https://kctc.kribb.re.kr

GDMCC Guangdong Microbial Culture Collection Center

Institute of Microbiology, Guangdong Academy of Sciences
TEL: (020) 87137633 E-mail: gdmcc@gdim.cn Web: http://www.gdmcc.net

Ref: 2025111

Certificate of Deposit

1. Name and address of the depositor or agent ：

Zhe Ma

Hospital of Stomatology Hebei Medical University

2. Day of issue: **September 15, 2025**

Accession number	Strain code by depositor(s)	Taxonomic designation
GDMCC 1.5953	I5G	*Moraxella* sp.
GDMCC 1.5954	XPZ19	*Streptococcus* sp.

All the above microorganisms have been successfully deposited into the general collection of microorganisms of the Guangdong Microbial Culture Collection Center (GDMCC) and confirmed the identity of the microorganisms under this GDMCC number. These strains are available in the public accessible section of the GDMCC and restrictions have not been placed on access. They will be included in the published and online catalogues after publication of this number by the authors.

Honghui Zhu

Honghui Zhu, Ph.D.
Director of GDMCC
Guangdong Microbial Culture Collection Center (GDMCC)
Institute of Microbiology, Guangdong Academy of Sciences
100 Xianlie Zhong Road, Yuexiu District, Guangzhou 510070, PR China
